# Fibronectin Adsorption on Electrospun Synthetic Vascular Grafts Attracts Endothelial Progenitor Cells and Promotes Endothelialization in Dynamic In Vitro Culture

**DOI:** 10.3390/cells9030778

**Published:** 2020-03-23

**Authors:** Ruben Daum, Dmitri Visser, Constanze Wild, Larysa Kutuzova, Maria Schneider, Günter Lorenz, Martin Weiss, Svenja Hinderer, Ulrich A. Stock, Martina Seifert, Katja Schenke-Layland

**Affiliations:** 1 NMI Natural and Medical Sciences Institute at the University of Tübingen, 72770 Reutlingen, Germany; ruben.daum@nmi.de (R.D.); dmitri.visser@nmi.de (D.V.); martin.weiss@med.uni-tuebingen.de (M.W.); hinderer@polymedics.de (S.H.); 2 Institute of Medical Immunology and BIH Center for Regenerative Therapies (BCRT), Charité-Universitätsmedizin Berlin, Corporate Member of Freie Universität Berlin, Humboldt-Universität zu Berlin, and Berlin Institute of Health, 13353 Berlin, Germany; constanze.wild@charite.de (C.W.); maria.schneider@charite.de (M.S.); martina.seifert@charite.de (M.S.); 3 Applied Chemistry, University of Reutlingen, 72762 Reutlingen, Germany; larysa.kutuzova@reutlingen-univeristy.de (L.K.); guenter.lorenz@reutlingen-university.de (G.L.); 4 Department of Women’s Health, Research Institute for Women’s Health, Eberhard-Karls-University Tübingen, 72076 Tübingen, Germany; 5 Department of Cardiothoracic Surgery, Royal Brompton and Harefield Foundation Trust, Harefield Hospital Hill End Rd, Harefiled UB9 6JH, UK; u.stock@rbht.nhs.uk; 6 Cluster of Excellence iFIT (EXC 2180) “Image-Guided and Functionally Instructed Tumor Therapies”, Eberhard-Karls-University Tübingen, 72076 Tübingen, Germany; 7 Department of Medicine/Cardiology, Cardiovascular Research Laboratories, David Geffen School of Medicine at UCLA, Los Angeles, CA 90095, USA

**Keywords:** vascular graft, endothelialization, tissue engineering, decorin, fibronectin, electrospinning, endothelial progenitor cells, bioreactor, biostable polyurethane

## Abstract

Appropriate mechanical properties and fast endothelialization of synthetic grafts are key to ensure long-term functionality of implants. We used a newly developed biostable polyurethane elastomer (TPCU) to engineer electrospun vascular scaffolds with promising mechanical properties (E-modulus: 4.8 ± 0.6 MPa, burst pressure: 3326 ± 78 mmHg), which were biofunctionalized with fibronectin (FN) and decorin (DCN). Neither uncoated nor biofunctionalized TPCU scaffolds induced major adverse immune responses except for minor signs of polymorph nuclear cell activation. The in vivo endothelial progenitor cell homing potential of the biofunctionalized scaffolds was simulated in vitro by attracting endothelial colony-forming cells (ECFCs). Although DCN coating did attract ECFCs in combination with FN (FN + DCN), DCN-coated TPCU scaffolds showed a cell-repellent effect in the absence of FN. In a tissue-engineering approach, the electrospun and biofunctionalized tubular grafts were cultured with primary-isolated vascular endothelial cells in a custom-made bioreactor under dynamic conditions with the aim to engineer an advanced therapy medicinal product. Both FN and FN + DCN functionalization supported the formation of a confluent and functional endothelial layer.

## 1. Introduction

Atherosclerotic cardiovascular disease is one of the leading causes of death worldwide [1,2]. It includes all medical conditions, where blood flow to organs and limbs is reduced due to plaque deposition. Surgical intervention is required to reopen or replace the defective vessel. The use of autografts, like the saphenous vein or mammary artery, are still the standard clinical approach for the replacement of small diameter blood vessels [3]. However, mechanical or size mismatches, and mainly the scarce availability make alternative grafts necessary [4,5]. In this context, two strategies have emerged in recent years: synthetic substitutes and biological grafts [4]. Although large-diameter synthetic substitutes (>6 mm) are successfully used, small diameter grafts (<6 mm) show low patency rates due to their tendency to elicit thrombosis and the formation of intimal hyperplasia [6,7,8]. Appropriate mechanical properties and biocompatibility of the synthetic graft as well as a fast endothelialization after implantation are key properties to ensure a long-term functional implant. In addition, the graft should evoke a balanced immune reaction. On the one hand, a moderate immune response is beneficial in order to promote tissue regeneration. On the other hand, chronic immune responses can lead to inflammation, fibrosis, or calcification and should be avoided to ensure long-term function of the vascular graft [9].

Electrospinning has proven to be a suitable method for the fabrication of fibrous scaffolds and vascular constructs as it mimics the highly porous structure and physical properties of the extracellular matrix (ECM) of the native tissue. Due to their high porosity, pore interconnectivity, and large surface area, the fibrous scaffolds are able to promote cell adhesion, cell alignment, and cell proliferation [10,11,12,13]. In addition, in order to elicit in situ endothelialization in the body, the material surface can be functionalized with bioactive molecules. A central challenge in this context is the attraction, adhesion, and proliferation of endothelial progenitor cells (EPCs) or endothelial cells (ECs) to form a complete endothelium. Several strategies to address this issue have been described: immobilization of antibodies targeting markers for EPCs such as vascular endothelial growth factor receptor 2 (VEGFR2) and platelet endothelial cell adhesion molecule (PECAM-1) [14,15]; modification of the surface with peptides such as the Arg-Gly-Asp (RGD) or Cys-Ala-Gly (CAG) sequence [16,17]; immobilization of growth factors such as the vascular endothelial growth factor (VEGF) or stromal cell-derived factor-1 (SDF-1) [18,19]; immobilization of oligonucleotides and aptamers [20,21]; and surface modification with oligosaccharides and phospholipids [22,23]. However, it is necessary to develop surfaces with improved biocompatible, bioactive, targeted, and stable biofunctionalization [24].

A recent study described the attraction of EPCs by immobilized recombinant human decorin (DCN) [25]. The small leucine-rich proteoglycan plays a pivotal role in the ECM [26]. It is named after its first known function as a modulator of collagen fibrillogenesis [27]. In recent years, it has been shown that DCN influences a variety of biological processes in addition to its structural function. It is involved in cell attachment [28,29,30], proliferation [31,32], and migration [28,29,31,33]. Furthermore, it has been described that DCN inhibits the proliferation and migration of vascular smooth muscle cells but does not affect ECs [28,31]. With a proportion of 22% of all proteoglycans in the vessel wall, it also influences many biological processes in vascular homeostasis and angiogenesis [34,35,36]. Depending on the molecular environment, it can act pro-angiogenic or antiangiogenic [26,34]. For instance, DCN was shown to interact antagonistically with the mesenchymal epithelial transition factor (c-MET) and the VEGFR2, which significantly influences angiogenesis [26,34,37,38]. In addition, DCN binds to the transforming growth factor β (TGF-β), which in turn has an inhibiting effect on the endothelial-mesenchymal transition and fibrosis [26,39,40]. These properties make the protein a promising candidate for improving the endothelialization of a vascular graft. Another highly relevant ECM protein is fibronectin (FN). Since FN interacts with cells via the integrins α_5_β_1_ or α_v_β_3_, it is a suitable protein for bioactivating a material surface [41,42,43,44]. It is of interest with regard to endothelialization, as it plays a pivotal role in wound healing [45,46]. Several studies described the coating of FN in combination with collagens type I [47] and type IV [48], with fibrinogen and tropoelastin [49], hepatocyte growth factor [50], heparin, and VEGF [51] and with SDF-1α [19] to improve reendothelialization. However, it has never been used in combination with DCN before.

Tissue engineering can be used as an alternative strategy to obtain a functional endothelium in a synthetic graft utilizing a patient’s own cells [52]. After implantation, the tissue-engineered vascular graft (TEVG) is replaced by the host’s cells and ECM and is thereby degraded [4]. However, the loss of mechanical properties due to a too rapid degradation and unfavorable biological reactions to the degradation products remain a major challenge [1,53]. A recent study addressed this problem by producing a TEGV that consists of a combination of a biodegradable and biostable polymer [54].

**Figure 1:**
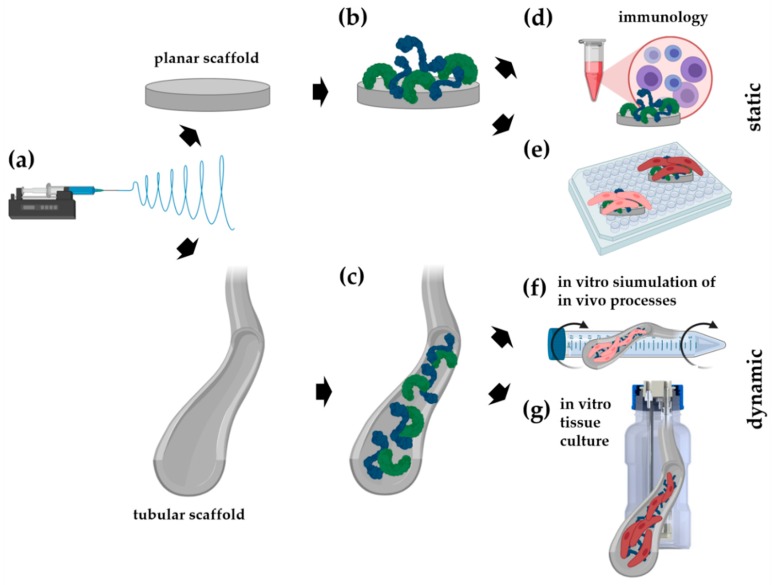
A newly developed polyurethane is used to produce planar and tubular electrospun scaffolds (**a**), which are biofunctionalized with either fibronectin (FN) or decorin (DCN) or with both extracellular matrix (ECM) proteins in combination (**b**,**c**). Besides investigating the immunology (**d**) and endothelial colony forming cell (ECFC) behavior on either planar (**e**) or in tubular scaffolds (**f**), the tubular scaffolds were also cultured with primary-isolated vascular endothelial cells (vECs) in an tissue-engineered vascular graft (TEVG) approach (**g**) in order to assess an ECM protein-improved endothelialization.

In our study, a newly developed biostable polyurethane elastomer was used to develop an electrospun scaffold with mechanical properties that are comparable to native vascular tissues, and a bioactive surface that attracts endothelial progenitor cells or promotes endothelialization [55]. For this purpose, planar and tubular electrospun scaffolds (Figure 1a) were biofunctionalized with FN, DCN, or FN and DCN in combination (FN + DCN; Figure 1b,c). The influence of the FN- and DCN-coated scaffolds on human immune cell features was examined (Figure 1d). Subsequently, the functionality of the electrospun scaffolds was further investigated. First, endothelial progenitor cell homing was simulated in vitro by attracting endothelial colony forming cells (ECFCs) with a potent angiogenic capacity and the capability to support vascular repair (Figure 1e,f). Secondly, in a classical TEVG approach primary-isolated vascular endothelial cells (vECs) were cultured in a custom-made bioreactor to create an advanced therapy medicinal product (ATMP) (Figure 1g).

## 2. Materials and Methods

### 2.1. Electrospun Scaffold Fabrication

Planar and tubular scaffolds were produced by electrospinning of soft thermoplastic polycarbonate-urethane (TPCU). This elastomeric material was synthesized in our laboratory for special medical applications using the multistep one-pot approach [56], which gives good control of the polymer architecture in catalyst-free systems. In more detail, a long-chain aliphatic polycarbonate with more than 72% (*w/w*) in the TPCU formulation provides an additional crystallization of the soft segment, which enhances biostability of the implantable material as well as improves its mechanical properties. In vitro biostability of the TPCU was studied previously from a mechanical point of view under long-term oxidative treatment [55]. Cytocompatibility of the TPCU material was also demonstrated [57]. By adjusting the respective parameters to achieve a stable process and appropriate mechanical properties of the scaffold (Figure S1a), 0.1 g/mL of the polymer was dissolved in 1,1,1,3,3,3 hexafluoro-2-propanol (804515, Merck, Darmstadt, Germany) and electrospun with the process conditions summarized in Table 1. The electrospinning process was carried out in a temperature- and humidity-controlled electrospinning apparatus (EC-CLI, IME Technologies, Eindhoven, Netherlands).

### 2.2. Biofunctionalization of the Scaffolds

Before biofunctionalization, the appropriate disinfection method was investigated. Since ethanol did not affect the scaffold in terms of its mechanical properties (Figure S1b), the constructs were disinfected with 70% ethanol for 20 min and afterwards washed three times for 10 min with phosphate-buffered saline (PBS). Microbiological studies were carried out on the scaffolds to investigate the effectiveness of the disinfection method (Figure S3). The scaffolds were functionalized by protein adsorption. They were incubated for 2 h at 37 °C with 20 µg/mL human plasma FN (F1056, Sigma-Aldrich, St. Louis, USA) or 20 µg/mL recombinant full-length human DCN [25], individually or in combination. Excess protein was removed by washing the scaffolds with PBS.

### 2.3. Morphological and Mechanical Characterization of the Electrospun Scaffolds

For the morphological characterization, punches from the electrospun scaffolds were examined by scanning electron microscopy (SU8030, Hitachi, Tokyo, Japan) followed by the analysis using ImageJ and the DiameterJ package [58] to assess the pore and fiber sizes. For the investigation of the mechanical properties, a ring tensile test was performed based on the methods described by Laterreur et al. [59] in order to determine the circumferential tensile strength and burst pressure. Briefly, the tubular scaffolds were cut into pieces with the length *L*_0_ = 7 mm, clamped into a uniaxial tensile testing device (Zwick Roell, Ulm, Germany), and stretched over a distance s with a velocity of 50 mm/min until rupture. On the basis of the stress–strain curves (Figure S1c), the burst pressure *P*_b_ was then calculated by relating the registered force at rupture *F_b_* to the elongation *s_b_* as follows:

(1) $$P_{b}=\frac{F_{b}\pi }{L_{0}d_{pin}\left(\pi +2\right)+2L_{0}s_{b}}$$

where *d*_pin_ represents the diameter of the pins that were used in the ring tensile test. A derivation of Equation (1) is provided by Lattereur et al. [59]. Using an OCA40 (DataPhysics Instruments GmbH, Filderstadt, Germany), the wettability of the scaffolds was analyzed as previously described [60]. A waterdrop with a volume of 2 µL was placed onto the scaffold and measured using the SCA20 software (DataPhysics Instruments, Filderstadt, Germany). The water absorption ability was determined by weighing the specimens in their dry and wet states after submerging the specimens in water for 1 h. The relative weight increase is referred to as the swelling ratio.

### 2.4. Immune Cell/Scaffold Co-Culture Assays

Polymorph nuclear cells (PMNs) were isolated from freshly donated human blood and peripheral blood mononuclear cells (PBMCs) from buffy coats according to the ethical approval by the local ethics committee at the Charité Berlin (EA2/139/10 approved on 10th December 2010; EA1/226/14 approved on 24th July 2014) and as recently described [61]. Monocytes were magnetically sorted via CD14 beads (130-050-201, Miltenyi Biotec, Bergisch Gladbach, Germany) from PBMCs as previously described [62]. Monocytes were differentiated into M0 macrophages by adding 50 ng/mL of macrophage colony-stimulating factor (M-CSF) (130-096-491, Miltenyi Biotec) to the culture medium for 7 days. All immune cell co-cultures were performed in Roswell Park Memorial Institute (RPMI) 1640 medium (F1415, Biochrom GmbH, Berlin, Germany) with 10% human serum type AB (H4522, Sigma-Aldrich), 1% L-glutamine (25030-024, Thermo Fisher Scientific, Waltham, MA, USA), and 1% penicillin/streptomycin (15140-122, Thermo Fisher Scientific).

First, the scaffold punches were incubated with 100 μg/mL of recombinant full-length human DCN [25] or 20 μg/mL of FN (F1056, Sigma-Aldrich) at 37 °C for 4 h. Next, punches were washed with PBS (L1825, Biochrom GmbH), placed into a well of a 48-well plate, and kept in place with a silicon ring (Ismatec, Wertheim, Germany). Thereafter, the different immune cell types were applied as follows:

Human PMNs were cultured on the uncoated, DCN- or FN-coated scaffolds; 0.2 × 10^6^ PMNs in 200 μL of complete RPMI were seeded directly on the scaffold punches. Unstimulated cells were used as a negative control, and PMNs that were stimulated with 500 ng/mL of lipopolysaccharide (LPS; 297-473-0, Sigma-Aldrich) served as a positive control. LPS is a component of the bacterial cell membrane that triggers the activation of immune cells. After 4 h of culture, cells were harvested only by careful resuspension, stained with human-specific antibodies for CD11b (1:100; 557701, BD Bioscience, San Jose, CA, USA) and CD66b (1:200; 305107, BioLegend, Fell, Germany), and measured by flow cytometry (CytoFLEX LX, Beckman Coulter, Inc., Brea, CA, USA) as described recently [61]. The determined mean fluorescence intensities (MFIs) of marker expression were normalized to the MFI of unstimulated PMNs directly after isolation.

Human monocytes or M0 macrophages were cultured on the uncoated, DCN- or FN-coated scaffolds; 0.2 × 10^6^ cells in 350 μL of complete RPMI were seeded directly on the scaffold punches. Monocytes that were stimulated with 100 ng/mL of LPS served as a positive control, and unstimulated monocytes served as a negative control. Macrophages cultured without any stimulus were used as negative control. To induce the polarization into the M1 phenotype, 20 ng/mL of IFNγ (130-096-486, Miltenyi Biotec) and 100 ng/mL of LPS were added to the medium of M0 macrophages. After two days of culture, monocytes/macrophages were harvested, stained with human-specific antibodies for CD80 (1:20; 305208, BioLegend) and human leukocyte antigen DR isotype (HLA-DR) (1:200; 307616, BioLegend), and measured by flow cytometry. Cells were detached by adding 100 μL of Accutase (A11105-01, Thermo Fisher Scientific) and incubating the cells at 37 °C for 30 min. The determined MFIs of the marker expression were normalized to the MFI of the unstimulated cells.

PBMCs were cultured on the uncoated, DCN- or FN-coated scaffolds; 0.3 × 10^6^ cells were seeded in 400 μL of complete RPMI directly on the scaffold punches. Unstimulated PBMCs served as a negative control. For the positive controls, PBMCs were stimulated with anti-CD28 (556620, BD Bioscience)/anti-CD3 (OKT3, Janssen-Cilag, Neuss, Germany) antibodies. After three days of culture, PBMCs were harvested, stained with human-specific antibodies for CD69 (1:50; 310926 BioLegend), CD25 (1:50; 302605, BioLegend) and HLA-DR (1:100; 307640, BioLegend), and measured by flow cytometry. PBMCs were detached by adding 100 μL of Accutase and by incubating the cells at 37 °C for 30 min. After gating for single and living cells the CD14− and CD14+ populations were defined. For CD3+ cells, the MFI of the activation markers CD25, CD69, and HLA-DR was determined. The determined MFIs of the marker expression were normalized to the MFI of unstimulated PBMCs.

Co-culture supernatants of monocytes and macrophages were collected and the tumor necrosis factor alpha (TNFα) concentration was analyzed by ELISA (430205, BioLegend) according to the manufacturer´s instructions.

### 2.5. Cell Culture of Primary Endothelial Cells and Endothelial Colony Forming Cells

Human primary-isolated vECs were isolated from foreskin biopsies under the ethics approval no 495/2018BO2 by enzymatic digestion with dispase and trypsin as previously described [63]. The vECs were cultured in endothelial cell growth medium and SupplementMix (C-22020, PromoCell, Heidelberg, Germany), supplemented with 1% penicillin-streptomycin (15140122, Thermo Fisher Scientific).

Human ECFCs (00189423, Lonza, Basel, Switzerland) were cultured in endothelial cell growth medium-2 with supplements (CC-3162, Lonza). Instead of the supplied fetal bovine serum, 5% of human serum (H4522, Sigma-Aldrich) was used. In addition, 1% L-Glutamine (21051024, Thermo Fisher Scientific) and 1% penicillin-streptomycin (15140122, Thermo Fisher Scientific) were added to the cell culture medium.

Both cell types were cultured at 37 °C and 5% CO_2_ and passaged at approximately 80% confluence. The vECs were used for the experiment after 2–4 passages.

### 2.6. Cell Seeding and Culture on Planar Scaffolds

Prior to cell culture experiments, general biocompatibility of the electrospun scaffolds was examined with a cytotoxicity test based on EN ISO 10993-5 [64]. Briefly, the scaffolds were incubated for 72 h at 37 °C and 5% CO_2_ in 1 mL endothelial cell growth medium supplemented with 1% penicillin-streptomycin at an extraction ratio of 0.1 mg/mL; 2 × 10^4^ vECs seeded in a 96-well plate were then exposed for 24 h to the extracts supplied with the cell culture medium supplements. Endothelial cell growth medium without the scaffolds served as a negative control. Cells exposed to 1% SDS served as positive control. The extraction and control medium were removed, and an MTS (3-(4,5-dimethylthiazol-2-yl)-5-(3-carboxymethoxyphenyl)-2-(4-sulfophenyl)-2H-tetrazolium) assay (CellTiter 96Aqueous One Solution Cell Proliferation Assay, Promega, Madison, WI, USA) was performed according to the manufacturer’s protocol; 20 µL MTS solution and 100 µL cell culture medium were added to each well. After 30 min of incubation at 37 °C, the absorbance of each well was measured at 450 nm using a microplate reader (PHERAstar, BMG Labtech, Ortenberg, Germany). Cell viability was determined by the absorbance of the samples relative to the negative control. No toxic effect of the material was observed (Figure S2a). Biofunctionalization of the scaffolds was then carried out as described above. Cells were seeded afterwards onto the biofunctionalized scaffolds with a diameter of 6 mm, which were placed in a 96-well plate. For the vECs, 5 × 10^3^ cells/well and, for the ECFCs, 1 × 10^4^ cells/well were seeded in 150 µL of the appropriate medium. If required, media change was carried out every 3 days.

### 2.7. Endothelial Colony Forming Cells (ECFC) Seeding Under Dynamic Conditions

The tubular electrospun scaffolds were cut to 6 cm length and biofunctionalized with FN and DCN alone or in combination as described above. A cell suspension of 4 × 10^5^ ECFCs/mL was pipetted into the tubular constructs. Afterwards, the constructs were closed at both ends and put in 15-mL centrifuge tubes filled with the corresponding cell culture medium. Placed on a roller mixer (RM5, CAT, Ballrechten-Dottingen, Germany), the tubes were rotated with 60 rpm for 24 h at 37 °C and 5% CO_2_. For cell number analysis, the attached cells were stained with 4′,6-diamidino-2-phenylindole (DAPI) (1:50, 10236276001, Roche Diagnostics, Mannheim, Germany) and counted.

### 2.8. Development of a Bioreactor System for Tissue-Engineered Vascular Graft (TEVG) Culture

The TEVG approach was performed with a custom-made bioreactor setup. The culture chamber consists of a 250-mL glass bottle (Schott Duran, Wertheim, Germany) and encloses a removable custom-designed graft frame that holds the vascular graft. A computer-aided design (CAD) model for the graft frame was created in Solidworks (Dassault Systèmes, Vélizy-Villacoublay, France) and milled out of polyether ether ketone (PEEK; ADS Kunststofftechnik, Ahaus, Germany) using a 2.5-axis flatbed milling setup (Isel, Eichenzell, Germany) with computer numerical control (CNC). The constructed parts were subjected to the aforementioned cytotoxicity test to ensure no toxic leachables are released into the medium under culture (Figure S2b). The modular design of the culture chamber allows for a toolless assembly of the bioreactor system under a sterile bench.

**Figure 2:**
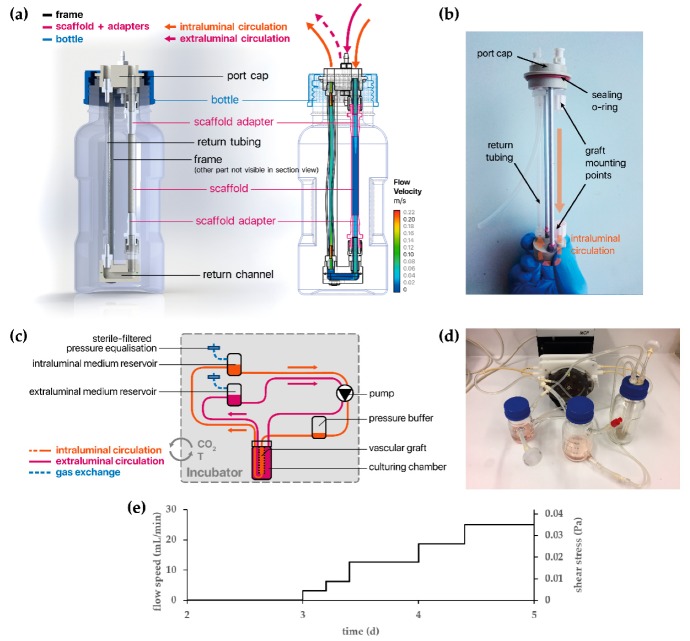
(**a**) A cross-sectional schematic representation of the culturing chamber and its parts. The wireframe model on the right is overlaid by the results of an in silico simulation and shows the flow velocity when the system is perfused with a flow rate of Q = 20 mL/min. (**b**) This photograph shows the graft frame (without scaffold), once it is taken out of the culturing chamber. (**c**) A schematic representation of the entire bioreactor setup, showing the circulation and connections to the medium reservoirs and pressure buffer/bubble trap. (**d**) A photograph showing the assembled bioreactor setup with all the components for the intraluminal circulation. (**e**) Applied perfusion flow speed as function of time with the corresponding wall shear stress.

The graft frame—once inserted into the culture chamber—is connected to medium reservoirs and a bubble trap with flexible silicone tubing. Sterile gas exchange is facilitated by sterile filters connected to the medium reservoirs. The entire setup is driven by a multichannel roller pump (Ismatec) (Figure 2). 

The flow rates *Q* for dynamic culture were determined with a derived formulation of the Hagen–Poiseuille equation for laminar flow in straight circular pipes with internal radius *r*:

(2) $$\tau =\frac{4\mu Q}{\pi r^{3}}$$

where *µ* denotes the dynamic viscosity. This gave an analytical approximation of the achieved wall shear stress (*τ*) within the cultured vascular graft. To validate this approximation and the assumption of a laminar regime within the vascular graft, in silico simulations were used to assess the local fluid dynamics within the vascular graft and graft frame interior. Briefly, the CAD model of the graft frame was meshed and exported to a computational fluid dynamics (CFD) solver (ANSYS Fluent). Dynamic culture with a wide range of flow rates was simulated under steady-state flow and Newtonian rheological conditions, after which the calculated wall shear stress on the interior graft wall was analyzed and compared to the aforementioned analytical solution (Figures S4 and S5).

### 2.9. Tissue Culture of Vascular Endothelial Cells Under Dynamic Conditions

Tubular electrospun scaffolds were cut to 7.5 cm length and biofunctionalized with 20 µg/mL FN as described previously. After inserting the graft frame into the culture chamber, 2 × 10^6^ vECs/mL were seeded into the tubular scaffold. In order to achieve homogeneous cell adhesion across the entire tube, the culture chamber was placed horizontally and rotated every 15 minutes over 45 ° for 3 h at 37 °C and 5% CO_2_. The culture chamber was consecutively connected to the rest of the bioreactor setup and filled with 70 mL culture medium, supplemented with 1% penicillin-streptomycin and 1% PrimocinTM (ant-pm-1, InvivoGen, San Diego, CA, USA). The seeded cells were allowed to proliferate under static conditions during the first three days, after which the flow rate was slowly increased over the course of two days, as shown in Figure 2e. Subsequently, the tubular construct was cultured under constant flow for seven days.

### 2.10. Immunofluorescence Staining

In order to examine the protein coating, the biofunctionalized scaffolds were stained using DCN mouse monoclonal IgG_1_ (1:200; sc-73896, Santa Cruz Biotechnology, Dallas, TX, USA) and FN polyclonal rabbit IgG (1:500; F3648, Sigma-Aldrich) antibodies. For fluorescence labeling, AlexaFluor 488 anti-mouse IgG (1:250; A-11001, Thermo Fisher Scientific) and AlexaFluor 546 anti-rabbit IgG (1:250; A-11035, Thermo Fisher Scientific) were used as secondary antibodies.

Cells cultured on the scaffolds were stained as follows: after washing once with PBS, the cell-seeded scaffolds were fixed with 4% paraformaldehyde (P6148, Sigma-Aldrich). In order to reduce nonspecific binding, the samples were incubated with 2% goat serum-containing block solution for 30 min. Afterwards, the cells were incubated over night at 4 °C with the following antibodies: Vascular endothelial cadherin (VE-cadherin) monoclonal mouse IgG_2B_ (1:500, MAB9381, R&D systems, Minneapolis, MN, USA), VEGFR2 polyclonal rabbit IgG (1:75, ab2349, Abcam, Cambridge, UK), PECAM-1 monoclonal mouse IgG_1_ (1:100, sc-71872, Santa Cruz), von Willebrand factor (vWF) polyclonal rabbit IgG (1:200, A0082, Dako, Glostrup, Denmark), and vinculin monoclonal mouse IgG_1_ (1:500, MAP3574, Millipore, Burlington, MA, USA). F-actin was stained for 45 min in the dark with Alexa Fluor 647 Phalloidin (1:500, A22287, Thermo Fisher Scientific). Subsequently, samples were incubated with the appropriate secondary antibodies (AlexaFluor 488 anti-mouse IgG, AlexaFluor 546 anti-rabbit IgG, and AlexaFluor 488 anti-mouse IgG2b (all 1:250; Thermo Fisher Scientific)).

Finally, nuclei were stained with DAPI (1:50) for 15 min in the dark. Images were obtained by using a fluorescence microscope (Cell Observer, Carl Zeiss AG, Oberkochen, Germany).

### 2.11. Examination of the Cell Coverage on the Tubular Scaffolds

The cell coverage of the inner wall of the tubular constructs was investigated using MTT (3-(4,5-dimethylthiazol-2-yl)-2,5-diphenyltetrazolium bromide) (M2128-1G, Sigma-Aldrich). After culturing with vECs, the constructs were incubated for 20 min with 1 mg/mL MTT at 37 °C and 5% CO_2_. The insoluble purple formazan produced by the cellular reduction of MTT was then examined macroscopically.

### 2.12. Image Analysis

FN and DCN coating were quantified by measuring the relative pixel intensity (RPI) of the immunofluorescence images. To assess protein expression in the experiments, the area within a defined fluorescence intensity threshold was measured and normalized to the cell number. The cell count in the static experiments was quantified by counting the DAPI-stained cell nuclei per area. The quantification of the adherent ECFCs in the dynamic experiment was performed by measuring the DAPI-stained area normalized to the total area. All images were analyzed using ImageJ [58].

### 2.13. Scanning Electron Microscopy of Cells

Prior to SEM imaging of the scaffolds with cells, a critical point drying step was performed. First, cells were fixed for 60 min with 4% paraformaldehyde (PFA)/ 25% glutaraldehyde in PBS. Subsequently, a series of ethanol solutions in ascending concentration up to 100% was carried out to remove water. Critical point drying was done with a CPD 030 (Bal-Tec AG, Balzers, Liechtenstein) according to the manufacturer’s protocol. Prior to imaging, the specimens were platina-coated (SCD050, Bal-Tec AG) for one minute at 0.05 mbar and rinsed with Argon after the coating process. SEM imaging was performed with a SU8030 (Hitachi, Tokyo, Japan) and an Auriga^®^ 40 (Zeiss, Oberkochen, Germany).

For SEM imaging of the monocytes and macrophages, the cells were cultured for two days on uncoated (w/o), DCN- or FN-coated scaffolds, followed by preparation (as described in Reference [62]) and imaging with a JCM 6000 Benchtop (JEOL, Freising, Germany).

### 2.14. Statistical Analysis

Except stated otherwise, data are presented as mean ± standard deviation. For the immune data, GraphPad Prism (GraphPad Software, San Diego, CA, USA) was used to determine statistical significance between two groups using a one-way ANOVA/Kruskal–Wallis test. For the other data, a one-way ANOVA/Fisher’s Least Significant Difference test was performed. A Welch’s t-test was performed to compare between two data groups using OriginPro (OriginLab, Northampton, MA, USA). Probability values of 95%, 99%, 99.9%, and 99.99% were used to determine significance.

## 3. Results

### 3.1. Biofunctionalization Does Not Impact the Mechanical Properties of Electrospun Tubular Constructs

**Figure 3:**
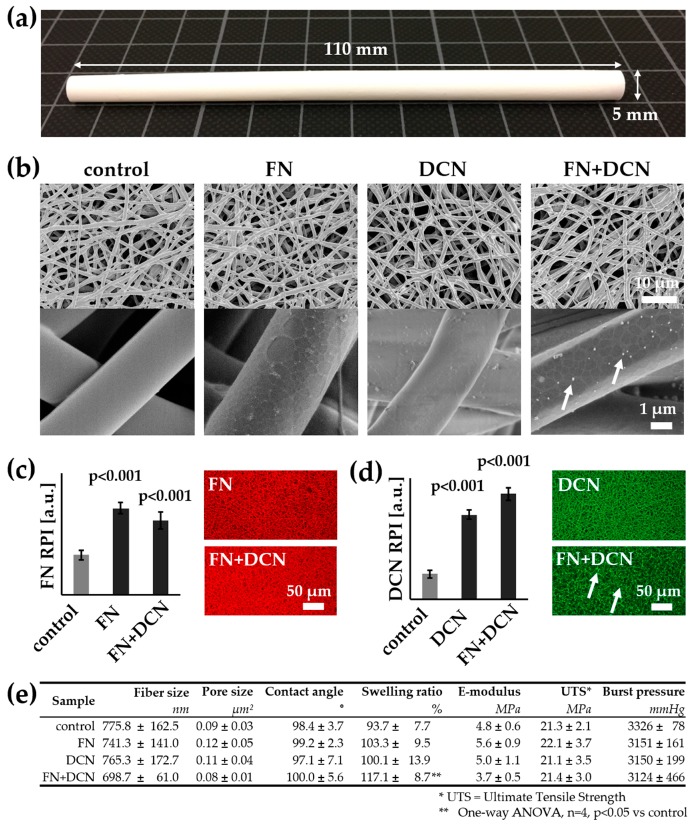
Morphological and mechanical characterization of the tubular biofunctionalized scaffolds: (**a**) Electrospun tubular scaffolds were fabricated with a length of 110 mm, an inner diameter of 5 mm, and a thickness of 0.40 ± 0.06 mm. (**b**) SEM images of control and biofunctionalized scaffolds: Scaffolds coated with FN show a network-like structure on the fibers. Aggregates deposited on the FN + DCN-coated samples are indicated by white arrows. (**c**,**d**) The coating of FN, DCN, or FN + DCN in combination was confirmed with IF staining: FN (red) and DCN (green). The white arrows indicate aggregates deposited on the FN + DCN-coated samples. Two-tailed *t*-test vs. control, n = 3, RPI = relative pixel intensity. (**e**) Fiber and pore size analysis shows no significant difference between the biofunctionalized scaffolds and the controls. Mechanical properties are not influenced by the protein coating. One-way ANOVA, n = 4, *p* < 0.05 vs. control.

Electrospinning was used to fabricate 110-mm long tubular scaffolds with an inner diameter of 5 mm and a thickness of 0.40 ± 0.06 mm (Figure 3a). In order to modulate the cell–material interaction, the surface was biofunctionalized with FN, DCN, or FN + DCN. The impact of the biofunctionalization on the morphological and mechanical properties of the material was investigated (Figure 3). Fiber and pore size analysis of the SEM images revealed no significant alteration due to protein adsorption (Figure 3e). Higher magnifications of the SEM images showed distribution of the proteins on the fibers. While DCN formed randomly distributed aggregates on the TPCU scaffolds, FN coating showed a network-like deposition in the nanometer range, which was also seen in the FN + DCN-coated samples, in which clearly recognizable aggregates were deposited on the protein network (Figure 3b, white arrows). Biofunctionalization utilizing both proteins individually and in combination was confirmed by IF staining. DCN IF staining revealed a more heterogeneous distribution of DCN in combination with FN than alone (Figure 3c, white arrows). The contact angle of the scaffolds was not significantly changed by the adsorption of either FN or DCN in comparison with the uncoated scaffolds. A significantly higher swelling ratio was observed of scaffolds that had been coated with FN + DCN (Figure 3e; control: 93.7% ± 7.7% versus FN + DCN: 117.1% ± 8.7%, *p* < 0.05). Overall, biofunctionalization had no significant influence on the mechanical properties (Figure 3e). The ultimate tensile strength ranged from 21.1 ± 3.5 MPa (DCN) to 22.1 ± 3.7 MPa (FN). Burst pressures were in the range between 3124 ± 466 mmHg (FN + DCN) to 3326 ± 78 mmHg (controls). Interestingly, the elastic modulus of the samples coated with FN + DCN showed a lower value compared to the controls, although this was not statistically significant (3.7 ± 0.5 MPa FN + DCN versus 4.8 ± 0.6 MPa controls, *p* = 0.125).

We compared the mechanical properties (elastic modulus and burst pressure) of our electrospun scaffolds with autologous grafts, which are today’s gold standard for vascular bypass surgeries, using data obtained from literature (Table 2) [65]. The elastic modulus of our constructs (4.8 ± 0.6 MPa) was slightly higher than that of saphenous veins (2.25–4.2 MPa) [66,67] and of iliofemoral arteries (1.54 MPa) and veins (3.11 MPa) [68]. However, compared with an internal mammary artery (8 MPa) and a femoral artery (FA, 10.5 MPa)—used for popliteal bypass surgery—our engineered scaffolds showed a lower elastic modulus [66,69,70]. Regarding the burst pressure, engineered scaffolds (3326 ± 78 mmHg) lied within the range of a saphenous vein (1250–3900 mmHg) [66,67,71,72] and an internal mammary artery (2000–3196 mmHg) [66,71]. Konig et al. recommends for a TEGV a minimum burst pressure of 1700 mmHg [71]. We can therefore argue that our constructs have suitable mechanical properties to serve as a vascular graft or TEGV.

### 3.2. Decorin and Fibronectin Coating of the Scaffolds Does Not Induce a Disadvantageous Immune Response

**Figure 4:**
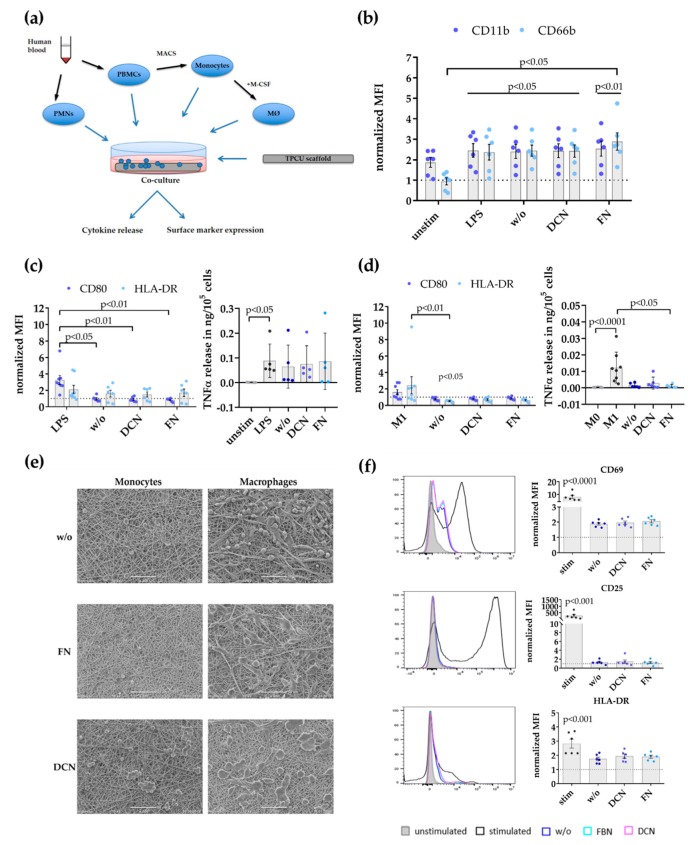
Immune response profile of FN- and DCN-coated planar scaffolds: (**a**) Schematic overview of the analysis steps and used immune cell assays. Polymorph nuclear cells (PMNs) and peripheral blood mononuclear cells (PBMCs) were isolated from human blood. Monocytes were acquired from PBMCs by magnetic separation via CD14 beads. Monocytes were differentiated into M0 macrophages (MØ) by stimulation with 50 ng/mL of macrophage colony-stimulating factor (M-CSF) for 7 days. (**b**) Surface expression of activation markers CD11b and CD66b by PMNs after 4 h: Displayed are the mean fluorescence intensities (MFI) normalized to unstimulated PMNs after isolation as mean ± SEM (standard error of the mean) for unstimulated (unstim) and lipopolysaccharide (LPS)-stimulated cells, as well as PMNs cultured on the uncoated (w/o), DCN-coated (DCN), and FN-coated (FN) scaffolds determined with flow cytometry. Kruskal–Wallis test, n = 6. (**c**) Surface expression of activation markers CD80 and human leukocyte antigen DR isotype (HLA-DR), and tumor necrosis factor alpha (TNFα) release by monocytes. Shown are the MFI normalized to unstimulated monocytes as mean ± SEM for LPS-stimulated cells as well as monocytes cultured on uncoated (w/o), DCN-coated (DCN), and FN-coated (FN) scaffolds. Kruskal–Wallis test, n = 6–8. The TNF release is depicted in ng/10^5^ cells as mean ± SEM for unstimulated (unstim) and LPS-stimulated cells as well as monocytes cultured on the uncoated (w/o), DCN-coated (DCN), and FN-coated (FN) scaffolds. Kruskal–Wallis test, n = 5. (**d**) Surface expression of activation markers CD80 and HLA-DR, and TNFα release by macrophage: Displayed is the MFI normalized to unstimulated M0 macrophages as mean ± SEM for macrophages differentiated to M1 and as well as cells cultured on uncoated (w/o), DCN-coated (DCN), and FN-coated (FN) scaffolds. Kruskal–Wallis test, n = 6–8. The TNFα release is shown in ng/10^5^ cells as mean ± SEM for unstimulated M0 macrophages; macrophages differentiated to M1; and as well as cells cultured on the uncoated (w/o), DCN-coated (DCN), and FN-coated (FN) scaffolds. Kruskal–Wallis test, n = 6–9. (**e**) Representative SEM images of monocytes (left) and macrophages (right) on uncoated (w/o) and with biofunctionalized scaffolds (DCN and FN). Scale bars represent 50 μm. (**f**) Expression of activation markers CD69, CD25, and HLA-DR on CD3+ T cells in whole PBMC co-cultures: Shown are representative histograms (left) and the surface expression levels as MFI normalized to unstimulated T cells as mean ± SEM (right) for αCD3/αCD28-stimulated T cells (stim) as well as T cells cultured on uncoated (w/o), DCN-coated, and FN-coated scaffold. Kruskal–Wallis test, n = 6.

The effect of DCN- or FN-coated TPCU scaffolds on immune cells was investigated in order to estimate their suitability as vascular graft material. The immune response of a combination coating was not required as the immune system would not react differently to the presence of both proteins in one coating. The performed immunological evaluation followed the normal sequence of immune activation [9], starting with PMNs that are followed by monocytes, which differentiate into macrophages at the site of injury, and finally T cells that become activated (Figure 4a).

Initially, the expression of known PMN activation markers, the integrin CD11b, and the adhesion molecule CD66b was analyzed (Figure 4b). The normalized mean fluorescence intensity (MFI) for CD11b (stim 2.461 ± 0.3323, *p* = 0.0179; w/o 2.406 ± 0.3393, *p* = 0.0378; DCN 2.442 ± 0.3361, *p* = 0.0217; FN 2.549 ± 0.3644, *p* < 0.0090; all versus unstim 0 hours 1 ± 0) and CD66b (stim 2.372 ± 0.3875, *p* = 0.0453; w/o 2.448 ± 0.2728, *p* = 0.0414; DCN 2.431 ± 0.3041, *p* = 0.0453; FN: 2.893 ± 0.4239, *p* = 0.0073; all versus unstim 0 h 1 ± 0) was significantly increased on PMNs after LPS stimulation (positive control) and, after culture on the uncoated/coated scaffolds, compared to the level of PMNs directly after isolation (dotted line, set to 1). Additionally, PMNs on FN-coated TPCU scaffolds displayed a significantly higher CD66b expression compared with the unstimulated controls (FN 2.893 ± 0.4239 versus unstim 4 h 0.9438 ± 0.1723, *p* < 0.0345).

In a next step, monocyte responses were studied by flow cytometry analysis of the activation markers CD80 and HLA-DR (Figure 4c). The expression level for the co-stimulatory molecule CD80 was significantly upregulated only on LPS-stimulated monocytes compared with all other experimental groups (LPS 3.254 ± 0.5533 versus w/o 0.9592 ± 0.1342, *p* = 0.0143; versus DCN 0.8888 ± 0.1209, *p* = 0.0046; versus FN 0.8325 ± 0.08414, *p* = 0.0018). No significant differences in HLA-DR expression were detectable between the tested conditions. Additionally, no enhanced TNFα release of monocytes cultured on the uncoated/coated scaffolds was measured in contrast to a significantly elevated secretion in the LPS-stimulated controls compared to the unstimulated controls (LPS 0.08859 ± 0.03039 versus unstim 0.0005580 ± 0.0002111, *p* = 0.0228).

Then, macrophages (M0 type) generated in vitro by M-CSF were screened for signs of activation or polarization (Figure 4d). M0 (unstimulated) and M1 macrophages (IFNγ/LPS-stimulated) were used as control groups. Enhanced CD80 and HLA-DR expression and increase of TNFα secretion are hallmarks of pro-inflammatory M1 macrophages. There was no difference in the CD80 expression level between M0 macrophages (dotted line, set to 1) and all other experimental groups. The expression of HLA-DR by macrophages on uncoated scaffolds was significantly decreased compared with the M0 and M1 control settings (w/o 0.5220 ± 0.05753 versus M0 1 ± 0, *p* = 0.0106; versus M1 2.453 ± 1.040, *p* = 0.0049). Whereas M1 macrophages significantly elevated their TNFα release compared with M0 macrophages (M1 0.01229 ± 0.003333 versus M0 0.0002707 ± 0.00004142, *p* < 0.0001), no enhancement in pro-inflammatory cytokine release was measurable in all other experimental groups. Macrophages on the FN-coated scaffolds actually decreased their TNFα release compared with the M1 controls (FN 0.0009826 ± 0.0004063 versus M1 0.01229 ± 0.003333, *p* = 0.0432). Complementary to the analysis of changes in surface marker and pro-inflammatory cytokine release by monocytes and macrophages, scanning electron microscopy was applied to assess the effects of co-culture on their morphology (Figure 4e). Scanning electron microscopy images were taken after the cells were cultured for two days on the different scaffold groups. Monocytes and macrophages on the DCN-coated scaffolds formed clusters of preferentially rounded cells. Macrophages cultured on uncoated or FN-coated scaffolds displayed more diverse shapes in contrast with cells grown on the DCN-coated TPCU scaffolds.

The potential activation of T cells was determined by flow cytometry analysis of known activation markers CD69, CD25, and HLA-DR [74] after culturing complete human PBMCs on either uncoated or coated scaffolds (Figure 4f). However, only anti-CD3/anti-CD28 stimulated T cells (stim; positive control) significantly elevated the expression level for CD69 (stim 7.956 ± 1.319 versus unstim 1 ± 0, *p* < 0.0001), CD25 (stim 265.6 ± 101.5 versus unstim 1 ± 0, *p* = 0.0008), and HLA-DR (stim 2.824 ± 0.3099 versus unstim 1 ± 0, *p* = 0.0001) compared with the level of the unstimulated controls (dotted line, set to 1). No significant increase in T cell activation marker expression was observed in any other experimental group.

### 3.3. Simulation of Endothelial Progenitor Cell Homing Using Endothelial Colony Forming Cells

#### 3.3.1. ECFCs Show Altered VEGFR2 and PECAM-1 Expression Patterns on FN + DCN-Coated TPCU Scaffolds Under Static Culture Conditions

**Figure 5:**
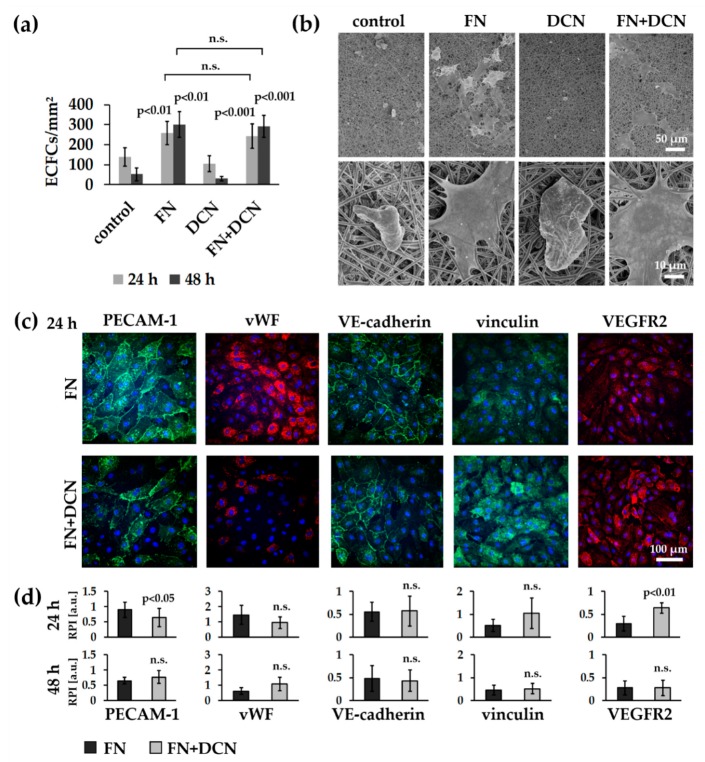
Static experiments of human ECFCs on FN-, DCN-, or FN + DCN-coated scaffolds: (**a**) Attachment and proliferation of the human ECFCs after 24 h and 48 h. Cells on FN and FN + DCN coating show a significantly higher proliferation when compared with cells gown on DCN and controls. Two-tailed *t*-test, compared to controls, n = 5, n.s. = not significant. (**b**) SEM images and (**c**) Immunofluorescence staining of ECFCs 24 h after seeding on ECM protein-coated scaffolds: Cells on FN and FN + DCN show a spread morphology in contrast to DCN coating and controls. (**d**) Semiquantitative fluorescence intensity analysis (relative pixel intensity (arbitrary units)) of cells on FN and FN + DCN shows no significant difference for the endothelial cell type marker von Willebrand factor (vWF) as well as vinculin and vascular endothelial cadherin (VE-cadherin). Platelet endothelial cell adhesion molecule (PECAM-1) expression is significantly decreased and VEGFR2 expression is significantly increased on FN + DCN-coated scaffolds after 24 h. Two-tailed *t*-test, n = 6, n.s. = not significant.

ECFCs were seeded on the biofunctionalized planar scaffolds and cultured under static conditions for 24 and 48 h. The amount of adherent ECFCs was significantly higher on samples coated with FN (24 h: 257 ± 57 cells/mm^2^ versus control with 137 ± 46 cells/mm^2^, *p* < 0.01; 48 h: 301 v 64 cells/mm^2^ versus control with 52 ± 32 cells/mm^2^, *p* < 0.001) and FN + DCN (24 h: 243 ± 63 cells/mm^2^ versus control with 137 ± 46 cells/mm^2^, *p* < 0.01; 48 h: 292 ± 54 cells/mm^2^ versus control with 52 ± 32 cells/mm^2^, *p* < 0.001) when compared with the uncoated samples (controls) throughout the entire culture period (Figure 5a). No significant difference of adherent cells was observed between FN coating and FN + DCN coating (24 h: *p* = 0.656; 48 h: *p* = 756). DCN coating did not show any significant difference in cell density in comparison with the uncoated controls (24 h: 105 ± 40 cells/mm^2^ versus control with 137 ± 46 cells/mm^2^, *p* = 0.340; 48 h: 30 ± 11 cells/mm^2^ versus control with 52 ± 32 cells/mm^2^, *p* = 0.460).

SEM analyses revealed that the ECFCs on the control and DCN-coated TPCU scaffolds had attained a spherical shape after 24 h whereas those on TPCU scaffolds that were coated with FN and FN + DCN showed a stretched morphology (Figure 5b). Immunofluorescence staining of samples 24 h after seeding (Figure 5c,d) identified a significantly lower PECAM-1 expression in ECFCs on FN + DCN-coated samples in comparison with FN coating (0.64 ± 0.30 versus 0.90 ± 0.25, *p* < 0.05). After 48 h, this effect tended to reverse, although the difference was not significant (0.70 ± 0.15 versus 0.54 ± 0.23, *p* = 0.073). A similar and statistically not significant tendency was detected for the fluorescence intensity of vWF. No significant changes were observed in VE-cadherin or vinculin expression. VEGFR2 expression was significantly decreased in cells cultured on FN-coated scaffolds when compared with cells grown on FN + DCN-coated scaffolds after 24 h (0.64 ± 0.11 versus 0.29 ± 0.16, *p* < 0.01). After 48 h, this effect vanished (0.28 ± 0.17 versus 0.28 ± 0.15, *p* = 0.942).

#### 3.3.2. FN + DCN-Coating Attracts ECFCs Under Dynamic Culture Conditions

**Figure 6:**
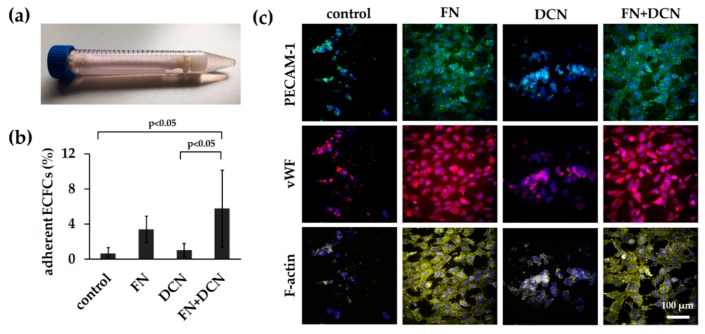
In vitro simulation of in vivo processes: ECFC attraction under dynamic conditions. (**a**) ECFCs were seeded into tubular constructs and cultured for 24 h on a roller mixer. (**b**) Adherent cells after 24 h on control scaffolds and on DCN-, FN-, and FN + DCN-coated scaffolds. FN + DCN coating shows a significantly higher cell number when compared with DCN coating and controls. One-way ANOVA, n = 4. (**c**) PECAM-1 (green), vWF (red), and F-actin (yellow) expression in ECFCs. Cells on FN and FN + DCN show a more spread morphology in contrast to the DCN and control samples.

After ECFC seeding under static conditions, the cell-seeded scaffolds were dynamically cultured on a roller mixer for 24 h (Figure 6a). This approach was performed to reflect more closely the in vivo conditions. The analysis of the adherent cells showed a significantly increased cell number on the FN + DCN-coated samples when compared with the controls and DCN-coated samples (5.7% ± 4.4% versus DCN coating with 1.0% ± 0.8%, *p* < 0.05 and versus control with 0.6% ± 0.7%, *p* < 0.05). The FN coating led to a nonsignificant decrease of adherent cells compared to FN + DCN coating (Figure 6b; 3.4% ± 1.5% versus 5.7% ± 4.4%, *p* = 0.226). Cells on all samples showed comparable PECAM-1 and vWF expression levels (Figure 6c). Distinct differences were observed in the cell morphology. F-actin staining helped visualizing the spread cells on the FN- and FN + DCN-coated scaffolds and cells with a more rounded morphology on the control samples and DCN-coated scaffolds (Figure 6c).

### 3.4. In Vitro Tissue Engineering Approach Using Vascular Endothelial Cells

#### 3.4.1. vECs Form an Endothelial Layer on FN- and FN + DCN-Coated Scaffolds Under Static Culture Conditions

**Figure 7:**
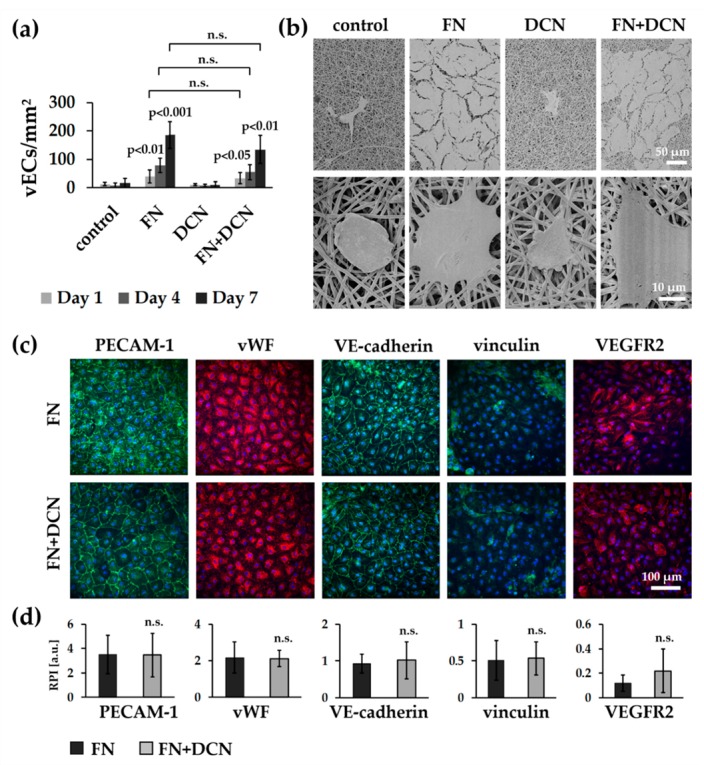
Static cell culture experiments of vECs on FN- and DCN-coated scaffolds: (**a**) Attachment and proliferation of vECs after 1, 4, and 7 days. vECs on FN and FN + DCN coating show a significantly higher proliferation rate compared with cells gown on DCN coating or control scaffolds. Two-tailed *t*-test, compared with control samples, n = 3, n.s. = not significant. (**b**) SEM images and (**c**) IF staining of vECs 7 days after seeding on ECM-coated scaffolds. Cells on FN and FN + DCN coating show a spread morphology in contrast with cells on DCN coating and control samples. (**d**) Semiquantitative fluorescence intensity analysis (relative pixel intensity (a.u.)) of cells on FN and FN + DCN coating shows no significant difference for PECAM-1, vWF, vinculin, or VE-cadherin expression. Two-tailed *t*-test, n = 5, n.s. = not significant.

vECs were seeded on the biofunctionalized planar constructs and cultured for 1, 4, and 7 days in order to investigate endothelialization (Figure 7a). One day after seeding, the cell number for all conditions was not significantly different. On day 4, vECs significantly increased proliferation on FN coating (78 ± 26 cells/mm^2^ versus control with 8 ± 7 cells/mm^2^, *p* < 0.01) and FN + DCN coating (55 ± 27 cells/mm^2^ versus control with 8 ± 7 cells/mm^2^, *p* < 0.05), while the VEC count on the DCN-coated samples had slightly decreased (7 ± 5 cells/mm^2^ versus control with 8 ± 7 cells/mm^2^, *p* < 0.931). This trend continued until day 7, on which a significantly increased cell count was detected for FN coating (186 ± 47 cells/mm^2^ versus control with 16 ± 16 cells/mm^2^, *p* < 0.001) and FN + DCN coating (135 ± 50 cells/mm^2^ versus control with 16 ± 16 cells/mm^2^, *p* < 0.01) in comparison with the uncoated controls. DCN coating of the TPCU scaffolds showed no improvement when compared with the control samples. Over the entire period of the experiment, the cell count was not significantly different between FN and FN + DCN coating.

While vECs on the control and DCN-coated scaffolds showed a spherical shape after 7 days as assessed using SEM, on FN and FN + DCN-coated scaffolds, vECs were stretched out and formed an almost confluent endothelial cell layer (Figure 7b). IF staining confirmed the expression of the endothelial cell type-specific markers PECAM-1, vWF, and VE-cadherin in the vECs on both FN and FN + DCN coating (Figure 7c). Semiquantitative analysis of fluorescence intensities revealed no significant differences of marker expression between FN and FN + DCN coating (Figure 7d). Vinculin expression was comparable in vECs on both coatings. With regard to VEGFR2, an increased fluorescence intensity in cells grown on the FN + DCN-coated samples was observed. However, due to a high variation in expression levels of individual experiments, no statistical significance between cells grown on FN or FN + DCN coating could be determined.

In summary, our data showed that DCN coating of the TPCU scaffolds did not have a substantial advantage when aiming for an increased VEC proliferation or an improved cell–cell or cell–material interaction. For this reason, only FN biofunctionalized TPCU scaffolds were used for the following in vitro tissue engineering experiments.

#### 3.4.2. vECs Cultured in a Custom-Made Bioreactor Under Flow Form a Confluent and Aligned Cell Layer on FN-Biofunctionalized TPCU

**Figure 8:**
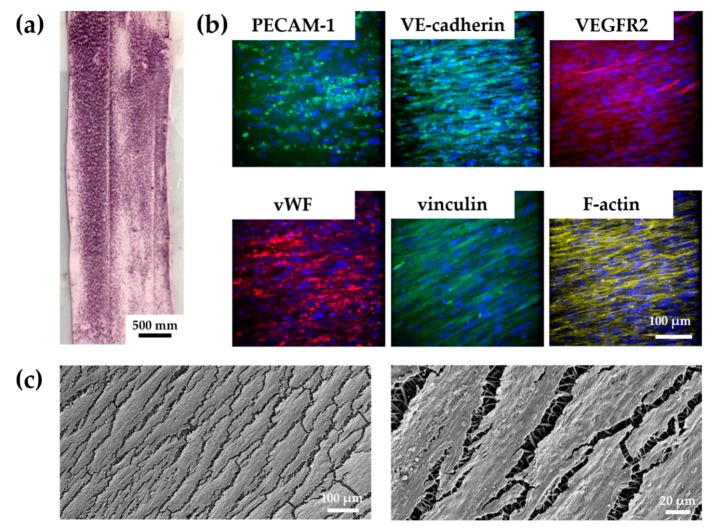
Tissue-engineering approach with vascular endothelial cells cultured for 7 days on FN-biofunctionalized electrospun tubular TPCU scaffolds under dynamic conditions: (**a**) Inner wall of the tubular construct shows living vECs indicated by the purple formazan stain. (**b**) PECAM-1, vWF, VE-cadherin, vinculin, VEGFR2, and F-actin expression were detected. vECs show an aligned morphology. (**c**) SEM confirms vECs that had aligned with the flow to which they were exposed to during the dynamic culture in the bioreactor.

After successful implementation of the developed bioreactor system, we aimed to test whether the FN-biofunctionalized TPCU scaffolds can be endothelialized under dynamic conditions. vECs were seeded into the tubular TPCU scaffolds, and after an initial culture for three days under static conditions to allow cell attachment, a flow was employed that was stepwise increased to 25 mL/min within 1.5 days (Figure 2e). Under this flow, which causes a shear stress of about 0.03 Pa, the vEC-seeded FN-biofunctionalized scaffolds were cultured for seven days. Metabolic activity assessment using an MTT assay showed that a large part of the inner wall of our construct was covered with living cells, as indicated by the purple formazan stain (Figure 8a). IF staining and SEM further revealed a layer of confluent vECs that were aligned in the direction of flow (Figure 8b,c).

We confirmed the expression of the endothelial cell markers PECAM-1, vWF, and VE-cadherin. However, PECAM-1 and VE-cadherin did not appear to be located on the cell membrane as usual. Vinculin and VEGFR2 were also detected in the cells. Nevertheless, the staining of VEGFR2 showed only a weak signal. 

## 4. Discussion

Due to a proven biocompatibility and biostability at body temperature [55,57], we selected for this study a novel thermoplastic polycarbonate urethane for the fabrication of a TEVG. At first, scaffolds were produced by electrospinning of the TPCU and were disinfected with 70% ethanol. Microbiological studies showed that ethanol treatment did not achieved 100% sterility (Figure S3; 2 out of 9 plates showed germ growth). We are aware that disinfection with ethanol does not necessarily inactivate all forms of microorganisms [75]; therefore, for the clinical translation, a more efficient sterilization method should be considered.

After disinfection, scaffolds were then biofunctionalized by adsorption of FN and DCN, either alone or in combination. The adsorbed proteins did not impact elastic modulus or burst pressure of the tubular constructs (Figure 3). We demonstrated that the biomechanical properties of our constructs were comparable to native vascular tissue (Table 2).

The ability to mimic the nanofibrous topography of the ECM makes electrospinning a powerful method for cardiovascular tissue-engineering applications. Several studies have already described the influence of fiber and pore size on cell adhesion, cell migration, proliferation, and differentiation, as well as cell–cell interaction [76,77,78]. In native blood vessels, the ECs are located on the basal lamina, a mixture of defined ECM proteins that form a network and bind cells [79]. The literature describes a wide range of pore and fiber diameters (1–1000 nm) from different vessels, depending on the position and physical properties of the vessel [80]. The main collagen component of the basal lamina is collagen type IV. It forms fibers that range from 20 to 52 nm [80,81,82]. In our study, the fiber diameters were between 699 ± 61 nm and 776 ± 163 nm, which is much higher compared to the collagen type IV fibers in native vessels. However, other studies developing electrospun vascular grafts reported comparable [83] or even larger fiber sizes [84,85] on which a functional endothelium was formed [84]. The pore size strongly depends on the vessel type and ranges between 5 nm and 8 µm [80,82,86,87,88,89]. Our constructs showed pore sizes between 0.08 ± 0.01 µm^2^ and 0.12 ± 0.05 µm^2^, which lies in the range of a native vessel.

Several studies have already described that FN improves the endothelialization of vascular grafts [19,48,49,51]. In our study, we observed a fibrous-like structure of the coated FN (Figure 3b). This phenomenon can be interpreted as material-driven fibrillogenesis, first described by Salmeron-Sanchez et al. [3]. In the human body, FN matrix assembly is a cell-mediated process [90] that influences cell growth, cell differentiation, and cell–cell interaction [76,77,78,90,91]. It has been shown that the adhesion of FN on poly (ethyl acrylate) (PEA) can lead to a spontaneous organization of FN into protein networks. It has also been shown that cell-free material-induced FN fibrillogenesis influences the maintenance and differentiation of stem cells [3,92]. Furthermore, it was described that the FN network has an increased ability to store growth factors [93]. To the best of our knowledge, our study is the first to show that material-driven fibrillogenesis can be observed on electrospun TPCU fibers. We presume that the surface properties, such as hydrophobicity and polarity, are comparable to those of PEA. Whether the FN network has a significant advantage in terms of cell behavior or growth factor binding compared to dispersed, coated FN molecules would need further investigation.

In addition to FN coating, in this study, we also used DCN coating. We observed that, after coating on the TPCU, DCN was randomly distributed in aggregates on the fibers (Figure 3b). Since DCN does not form fibrils, this coating behavior was expected. Even larger, globular DCN aggregates were observed on the FN + DCN samples (Figure 3b,d). Interestingly, these aggregates were predominantly seen on the FN fibrils and not on the TPCU itself. It is known that DCN interacts with FN [94,95]. Furthermore, the interaction of proteins with materials is determined by the geometrical, chemical, and electrical properties of the substrate [96]. In this respect, it can be hypothesized that the DCN prefers the FN surface more than the hydrophobic polyurethane surface. Interestingly, we observed a significantly increased swelling ratio for FN + DCN (Figure 3e). This was not the case with individually FN- or DCN-coated TPCU. Depending on the surface properties of the material and the interaction with other proteins, the conformation, orientation, and bioactivity of a protein can also be influenced [96,97,98]. With this in mind, one can assume that both DCN and FN in combination can have a different bioactivity [99].

In contrast to our previous findings using poly (ethylene glycol) dimethacrylate-poly (L-lactide) (PEGdma-PLA) or a blend of poly-ε-caprolacton and gelatin [25,100], we identified a cell-repellent effect of the DCN-coated TCPU electrospun scaffolds for both human ECFCs and human vECs. As already discussed, cells prefer to adhere to hydrophilic surfaces [101]. Since the TCPU itself is highly hydrophobic (control: 98.4 ± 3.7 °), it can cause a cell-repellent effect. DCN alone was not able to diminish this effect (Figure 5a,b). Cell adhesion is influenced by cell-adhesive peptides such as the RGD sequence. Since DCN does not contain these sequences, as it is the case with FN, we assume that at least this integrin-based cell–material interaction cannot be mediated by DCN. It has been described that DCN can even partially inhibit cell adhesion; however, this has only been observed with fibroblasts and not with endothelial cells [28,102]. Hinderer et al. observed an attraction of ECFCs to DCN-coated PEGdma-PLA [25]. A direct comparison with this study is therefore difficult, since this polymer has different surface properties, which influence the amount and orientation of the adsorbed DCN and thus may have an altered impact on cell behavior [96]. FN coating reversed the cell-repellent effect of the TCPU, both with and without DCN (Figure 5). We can therefore conclude that the cell attraction and proliferation is supported by FN but not affected by DCN [99,103].

Scaffolds should in general exhibit a low immunogenicity and at the same time support tissue regenerative processes. The evaluation of the immune response profiles of the analyzed control and ECM-coated scaffolds excluded any major adverse effects, with only minor innate activation characteristics. Co-culturing PMNs, as the first cells of an innate immune response, induced an activated cell phenotype regarding the expression of CD11b and CD66b. Monocytes were incompletely activated after co-culturing with the scaffold as indicated by only a weak tendency to upregulate the HLA-DR expression and to increase their TNFα release. From the literature, it is well known that the upregulation of CD80 and HLA-DR would be a hallmark of M1 macrophages [62,104] and that the fiber and pore size of electrospun scaffolds could impact the macrophage polarization state [105]. When analyzing the potential impact of the TPCU scaffolds on macrophage polarization, no clear trend to drive the process into a specific macrophage subtype could be determined. Also, the coating by either DCN or FN did not trigger a specific type of macrophage polarization. In contrast, co-culture studies with soluble recombinant DCN demonstrated that macrophages responded with an upregulated CD80 expression as well an increased secretion of TNFα and IL-10 [25]. The absent responses in the present study may result from the far lower amount of protein present on the coated scaffolds in comparison with the high protein amounts available within solutions or even by conformational changes. Not surprisingly, adaptive T cell responses were also not detected. T cells on scaffolds simply showed a trend to upregulate CD69 and HLA-DR without significant changes.

A functional endothelium is mainly characterized by cell–cell junctions [106]. As PECAM-1 is the most abundant component of the EC junction, which contributes to the maintenance of the EC permeability barrier, its expression is essential for a functional EC layer [107]. In our study, the ECFCs on FN coating revealed a significantly increased PECAM-1 expression after 24 h compared with ECFCs cultured on FN + DCN-coated scaffolds. In contrast, the VEGFR2 expression was significantly decreased in the ECFCs on FN coating after 24 h compared with FN + DCN coating. It has been reported that VEGFR2 is highly expressed in early endothelial precursor cells but not in all mature ECs [108,109]. For example, PECAM-1 is less expressed in endothelial progenitor cells, as it is typically associated with a more mature EC phenotype [110]. Interestingly, DCN has been reported to stimulate the maintenance of undifferentiated progenitor cells [111], and FN promotes endothelial cell differentiation [112]. Therefore, we hypothesize that the FN + DCN coating in our experiments kept the ECFCs in a precursor cell state compared with the culture on only FN. It may also be possible that a direct interaction of DCN with VEGFR2 leads to its upregulation. A positive feedback loop between VEGF and VEGFR2 has been described [113]. Whether DCN has the same effect remains to be confirmed.

Since DCN exerts many other functions, an indirect regulation of VEGFR2 is also conceivable [34,114]. Mazor et al. showed that the matrix metalloproteinase-1 (MMP-1) promotes the expression of VEGFR2 [115]. The core protein of DCN in turn is able to stimulate the expression of MMP-1 [116,117]. Furthermore, Murakami et al. reported that increased concentrations of the fibroblast growth factor (FGF) led to an increase in VEGFR2 levels [118]. DCN, in turn, can bind to FGF and can increase its activity [119]. It was also described that VEGFR2 expression is regulated by the disruption of the c-MET receptor tyrosine kinase [120]. As an antagonistic ligand of c-MET, DCN is able to inhibit its activity and thus might indirectly promote VEGFR2 expression [38]. We have already discussed the hypothesis that DCN in interaction with FN may exhibit an altered bioactivity. This would explain why DCN, which was adsorbed on the TPCU scaffold surface, impacted ECs in combination with FN but did not without [96,97,98]. The reason for VEGFR2 upregulation can also be due to FN. It might be possible that, in combination with DCN, its conformation and function is also changed [96,97,98]. It has been shown that conformational remodeling of the FN matrix selectively regulates VEGF signaling [121]. VEGF in turn regulates VEGFR2 expression [113]. By binding to VEGF, FN can promote full phosphorylation and activation of VEGFR2 [122]. Interestingly, after 48 h, the difference between FN and FN + DCN coating for both the PECAM-1 and VEGFR2 expression had vanished (Figure 5d). With regard to VEGFR2, a short half-life of the receptor is described, which enables ECs to adapt quickly to changes in the extracellular environment [118,123]. This leads to the question of how long the biofunctionalized DCN coating was fully biologically active in our study. Due to its natural presence in the body, it can be easily degraded [124]. We showed that DCN acts on ECFCs for at least 24 h under static conditions. The culture of vECs over 7 days under static conditions revealed the same expression of PECAM-1 and VEGFR2 on FN and FN + DCN coating (Figure 7). This observation supports the assumption that the DCN was only active for a short period of time and that its effect had disappeared after 7 days. In addition, it is possible that the vECs are not as sensitive to DCN, as we have observed with the ECFCs. Several studies have described an increase in VEGFR2 expression during differentiation and expansion of endothelial progenitor cells [109,125]. At the same time, VEGFR2 expression was relatively low during the proliferation phase [126]. Since the vECs are mature cells, it can be assumed that the externally changed conditions do not affect the VEFGR2 expression significantly. Nevertheless, in this study, we successfully showed that vECs formed an endothelium on biofunctionalized FN-coated constructs after 7 days of culture whereas DCN-coated TPCU scaffolds did not show a significant effect on cell proliferation.

In our TEVG experiments using a custom-made bioreactor, we observed a unidirectional cell orientation in the direction of the flow. The response of ECs to shear stress is well studied [127,128,129]. It has been shown that, under flow, the morphology of vECs changes from a cobblestone (static) to an elongated form and that vECs align in the direction of the flow in only 24 h [127]. The hemodynamic forces can modulate not only the phenotype but also the gene expression of the cells. In this context, the correct flow is of great importance for a properly functioning endothelium [130]. In our study, IF staining revealed the expression of vWF, PECAM-1, and VE-cadherin. However, PECAM-1 and VE-cadherin were not located on the cell membrane as usually seen. VEGFR2 expression was quite weak, and the F-actin staining revealed a rather fibroblast-like cell morphology. We hypothesize that the vECs underwent endothelial-mesenchymal transition (EndMT). ECs, which undergo EndMT, lose the expression of the characteristic surface endothelial markers PECAM-1, VE-cadherin, and VEGFR2 [39,131,132]. Mahmoud et al. showed that the EndMT can be induced under low shear stress (0.4 Pa) [133]. In our approach, the cells experienced a wall shear stress of about 0.03 Pa, which is slightly lower than a venous wall shear stress (0.06 Pa) [134]. In silico simulations of our dynamic bioreactor culture confirmed laminar flow conditions along a large part of the vascular wall using the applied parameters. Another reason for the fibroblast-like phenotype could be that ECs are highly plastic [135,136]. Therefore, culturing ECs in vitro in an artificial environment can lead to cell dedifferentiation [136,137]. This highlights the importance of fine-tuning the culture conditions to create a functional TEGV.

## 5. Conclusions

In the present study, we successfully engineered a TPCU electrospun vascular graft which combines appropriate mechanical properties with a highly bioactive surface for the attraction of ECs. The FN biofunctionalization was characterized by a material-driven fibrillogenesis, which might have a positive impact on FN functionality [3]. To imitate the physiological conditions of a blood vessel, a bioreactor for in vitro tissue culture was designed and manufactured. vECs seeded on the FN-functionalized constructs formed a confluent and functional endothelium under static and dynamic conditions. In contrast, DCN-biofunctionalized TPCU scaffolds had a cell-repellent effect on vECs and ECFCs, most likely due to the high hydrophobic properties of the TPCU. However, since DCN has been shown to inhibit the adhesion of fibroblasts, it remains a promising protein for the functionalization of vascular grafts [29].

The challenge for the future will be to combine the advantages of different proteins and to thus increase the selectivity, functionality, and stability of a biofunctionalized vascular graft while keeping the complexity of the coating as low as possible.

## Figures and Tables

**Table 1:** Process conditions for electrospinning planar and tubular scaffolds.

Description	Value
Distance	25 cm
Needle i.d.	0.4 mm
Voltage	18 kV/−0.2 kV (needle/collector)
Temperature	23 °C
Humidity	40%
Mandrel diameter ^1^	6 mm
Mandrel rotation speed ^1^	2000 rpm
Needle translation distance ^1^	80 mm
Volume	6 mL
Flow rate	4 mL/h

i.d.= inner diameter; ^1^ tubular scaffolds.

**Table 2:** Mechanical properties of the electrospun constructs and native blood vessels.

Graft Type	Elastic Modulus (MPa)	Burst Pressure (mmHg)	Ref.
Electrospun vascular graft	4.8 ± 0.6	3326 ± 78	-
Saphenous vein	4.2	1680–3900	[66]
Saphenous vein	2.25	1250	[67]
Saphenous vein	NA	1680	[73]
Saphenous vein	NA	2200	[72]
Saphenous vein	NA	1599	[71]
Internal mammary artery	NA	3196	[71]
Internal mammary artery	8	2000	[66]
Femoral artery	9–12	NA	[69]
Iliofemoral artery	1.54	NA	[68]
Iliofemoral vein	3.11	NA	[68]

## Supplementary Materials

The following are available online at https://www.mdpi.com/2073-4409/9/3/778/s1, Figure S1: Mechanical characterization of the electrospun TPCU scaffolds and biocompatibility of the materials, Figure S2: Cytotoxicity tests of the materials, Figure S3: Microbiological studies of the ethanol disinfected electrospun TPCU scaffolds, Figure S4: The part of the culture chamber that was considered for CFD simulations, Figure S5: The Poiseuille values (developed wall shear stress value) within the scaffold for different flow rates.

## References

[1] Catto V., Farè S., Freddi G., Tanzi M.C. (2014). Vascular Tissue Engineering: Recent Advances in Small Diameter Blood Vessel Regeneration. ISRN Vasc. Med..

[2] Causes of Death. https://www.who.int/data/gho/data/themes/topics/causes-of-death/GHO/causes-of-death.

[3] Salmerón-Sánchez M., Rico P., Moratal D., Lee T.T., Schwarzbauer J.E., García A.J. (2011). Role of Material-Driven Fibronectin Fibrillogenesis in Cell Differentiation. Biomaterials.

[4] Sánchez P.F., Brey E.M., Carlos Briceño J.C. (2018). Endothelialization Mechanisms in Vascular Grafts. J. Tissue Eng. Regen. Med..

[5] L’Heureux N., Dusserre N., Marini A., Garrido S., De la Fuente L., McAllister T. (2007). Technology Insight: The Evolution of Tissue-Engineered Vascular Grafts - From Research to Clinical Practice. Nat. Clin. Pract. Cardiovasc. Med..

[6] Ercolani E., Del Gaudio C., Bianco A. (2015). Vascular Tissue Engineering of Small-Diameter Blood Vessels: Reviewing the Electrospinning Approach. J. Tissue Eng. Regen. Med..

[7] Seifu D.G., Purnama A., Mequanint K., Mantovani D. (2013). Small-Diameter Vascular Tissue Engineering. Nat. Rev. Cardiol..

[8] Ravi S., Qu Z., Chaikof E.L. (2009). Polymeric Materials for Tissue Engineering of Arterial Substitutes. Vascular.

[9] Julier Z., Park A.J., Briquez P.S., Martino M.M. (2017). Promoting Tissue Regeneration by Modulating the Immune System. Acta Biomater..

[10] Hinderer S., Brauchle E., Schenke-Layland K. (2015). Generation and Assessment of Functional Biomaterial Scaffolds for Applications in Cardiovascular Tissue Engineering and Regenerative Medicine. Adv. Healthc. Mater..

[11] Ndreu A., Nikkola L., Ylikauppilar H., Ashammakhi N., Hasirci V. (2008). Electrospun Biodegradable Nanofibrous Mats for Tissue Engineering. Nanomedicine.

[12] Li M., Mondrinos M.J., Gandhi M.R., Ko F.K., Weiss A.S., Lelkes P.I. (2005). Electrospun Protein Fibers as Matrices for Tissue Engineering. Biomaterials.

[13] Boland E.D., Matthews J.A., Pawlowski K.J., Simpson D.G., Wnek G.E., Bowlin G.L. (2004). Electrospinning Collagen and Elastin: Preliminary Vascular Tissue Engineering. Front. Biosci..

[14] Zhang M., Wang Z., Wang Z., Feng S., Xu H., Zhao Q., Wang S., Fang J., Qiao M., Kong D. (2011). Immobilization of Anti-CD31 Antibody on Electrospun Poly(E{open}-Caprolactone) Scaffolds through Hydrophobins for Specific Adhesion of Endothelial Cells. Colloids Surf. B Biointerfaces.

[15] Markway B.D., McCarty O.J.T., Marzec U.M., Courtman D.W., Hanson S.R., Hinds M.T. (2008). Capture of Flowing Endothelial Cells Using Surface-Immobilized Anti-Kinase Insert Domain Receptor Antibody. Tissue Eng.-Part C Methods.

[16] Kanie K., Narita Y., Zhao Y., Kuwabara F., Satake M., Honda S., Kaneko H., Yoshioka T., Okochi M., Honda H. (2012). Collagen Type IV-Specific Tripeptides for Selective Adhesion of Endothelial and Smooth Muscle Cells. Biotechnol. Bioeng..

[17] Li J., Ding M., Fu Q., Tan H., Xie X., Zhong Y. (2008). A Novel Strategy to Graft RGD Peptide on Biomaterials Surfaces for Endothelization of Small-Diamater Vascular Grafts and Tissue Engineering Blood Vessel. J. Mater. Sci. Mater. Med..

[18] Edlund U., Sauter T., Albertsson A.-C. (2011). Covalent VEGF Protein Immobilization on Resorbable Polymeric Surfaces. Polym. Adv. Technol..

[19] De Visscher G., Mesure L., Meuris B., Ivanova A., Flameng W. (2012). Improved Endothelialization and Reduced Thrombosis by Coating a Synthetic Vascular Graft with Fibronectin and Stem Cell Homing Factor SDF-1α. Acta Biomater..

[20] Schleicher M., Hansmann J., Elkin B., Kluger P.J., Liebscher S., Huber A.J.T., Fritze O., Schille C., Müller M., Schenke-Layland K. (2012). Oligonucleotide and Parylene Surface Coating of Polystyrene and EPTFE for Improved Endothelial Cell Attachment and Hemocompatibility. Int. J. Biomater..

[21] Strahm Y., Flueckiger A., Billinger M., Meier P., Mettler D., Weisser S., Schaffner T., Hess O. (2010). Endothelial-Cell-Binding Aptamer for Coating of Intracoronary Stents. J. Invasive Cardiol..

[22] Suuronen E.J., Zhang P., Kuraitis D., Cao X., Melhuish A., McKee D., Li F., Mesana T.G., Veinot J.P., Ruel M. (2009). An Acellular Matrix-Bound Ligand Enhances the Mobilization, Recruitment and Therapeutic Effects of Circulating Progenitor Cells in a Hindlimb Ischemia Model. FASEB J..

[23] Tardif K., Cloutier I., Miao Z., Lemieux C., St-Denis C., Winnik F.M., Tanguay J.F. (2011). A Phosphorylcholine-Modified Chitosan Polymer as an Endothelial Progenitor Cell Supporting Matrix. Biomaterials.

[24] Melchiorri A.J., Hibino N., Fisher J.P. (2013). Strategies and Techniques to Enhance the in Situ Endothelialization of Small-Diameter Biodegradable Polymeric Vascular Grafts. Tissue Eng. Part B. Rev..

[25] Hinderer S., Sudrow K., Schneider M., Holeiter M., Layland S.L., Seifert M., Schenke-Layland K. (2018). Surface Functionalization of Electrospun Scaffolds Using Recombinant Human Decorin Attracts Circulating Endothelial Progenitor Cells. Sci. Rep..

[26] Zhang W., Ge Y., Cheng Q., Zhang Q., Fang L., Zheng J. (2018). Decorin Is a Pivotal Effector in the Extracellular Matrix and Tumour Microenvironment. Oncotarget.

[27] Chen S., Young M.F., Chakravarti S., Birk D.E. (2014). Interclass Small Leucine-Rich Repeat Proteoglycan Interactions Regulate Collagen Fibrillogenesis and Corneal Stromal Assembly. Matrix Biol..

[28] Fiedler L.R., Schönherr E., Waddington R., Niland S., Seidler D.G., Aeschlimann D., Eble J.A. (2008). Decorin Regulates Endothelial Cell Motility on Collagen I through Activation of Insulin-like Growth Factor I Receptor and Modulation of A2β1 Integrin Activity. J. Biol. Chem..

[29] Fiedler L.R., Eble J.A. (2009). Decorin Regulates Endothelial Cell-Matrix Interactions during Angiogenesis. Cell Adh. Migr..

[30] Zafiropoulos A., Nikitovic D., Katonis P., Tsatsakis A., Karamanos N.K., Tzanakakis G.N. (2008). Decorin-Induced Growth Inhibition Is Overcome through Protracted Expression and Activation of Epidermal Growth Factor Receptors in Osteosarcoma Cells. Mol. Cancer Res..

[31] Nili N., Cheema A.N., Giordano F.J., Barolet A.W., Babaei S., Hickey R., Eskandarian M.R., Smeets M., Butany J., Pasterkamp G. (2003). Decorin Inhibition of PDGF-Stimulated Vascular Smooth Muscle Cell Function: Potential Mechanism for Inhibition of Intimal Hyperplasia after Balloon Angioplasty. Am. J. Pathol..

[32] D’Antoni M.L., Risse P.A., Ferraro P., Martin J.G., Ludwig M.S. (2012). Effects of Decorin and Biglycan on Human Airway Smooth Muscle Cell Adhesion. Matrix Biol..

[33] De Lange Davies C., Melder R.J., Munn L.L., Mouta-Carreira C., Jain R.K., Boucher Y. (2001). Decorin Inhibits Endothelial Migration and Tube-like Structure Formation: Role of Thrombospondin-1. Microvasc. Res..

[34] Järveläinen H., Sainio A., Wight T.N. (2015). Pivotal Role for Decorin in Angiogenesis. Matrix Biol..

[35] Riessen R., Isner J.M., Blessing E., Loushin C., Nikol S., Wight T.N. (1994). Regional Differences in the Distribution of the Proteoglycans Biglycan and Decorin in the Extracellular Matrix of Atherosclerotic and Restenotic Human Coronary Arteries. Am. J. Pathol..

[36] Salisbury B.G., Wagner W.D. (1981). Isolation and Preliminary Characterization of Proteoglycans Dissociatively Extracted from Human Aorta. J. Biol. Chem..

[37] Khan G.A., Girish G.V., Lala N., di Guglielmo G.M., Lala P.K. (2011). Decorin Is a Novel VEGFR-2-Binding Antagonist for the Human Extravillous Trophoblast. Mol. Endocrinol..

[38] Goldoni S., Humphries A., Nyström A., Sattar S., Owens R.T., McQuillan D.J., Ireton K., Iozzo R.V. (2009). Decorin Is a Novel Antagonistic Ligand of the Met Receptor. J. Cell Biol..

[39] Van Meeteren L.A., Ten Dijke P. (2012). Regulation of Endothelial Cell Plasticity by TGF-β. Cell Tissue Res..

[40] Järvinen T.A.H., Ruoslahti E. (2010). Target-Seeking Antifibrotic Compound Enhances Wound Healing and Suppresses Scar Formation in Mice. Proc. Natl. Acad. Sci. USA.

[41] Liverani L., Killian M.S., Boccaccini A.R. (2019). Fibronectin Functionalized Electrospun Fibers by Using Benign Solvents: Best Way to Achieve Effective Functionalization. Front. Bioeng. Biotechnol..

[42] Campos D.M., Gritsch K., Salles V., Attik G.N., Grosgogeat B. (2014). Surface Entrapment of Fibronectin on Electrospun PLGA Scaffolds for Periodontal Tissue Engineering. Biores. Open Access.

[43] Regis S., Youssefian S., Jassal M., Phaneuf M., Rahbar N., Bhowmick S. (2014). Integrin A5β1-Mediated Attachment of NIH/3T3 Fibroblasts to Fibronectin Adsorbed onto Electrospun Polymer Scaffolds. Polym. Eng. Sci..

[44] Monchaux E., Vermette P. (2010). Effects of Surface Properties and Bioactivation of Biomaterials on Endothelial Cells. Front. Biosci.-Sch..

[45] Lenselink E.A. (2015). Role of Fibronectin in Normal Wound Healing. Int. Wound J..

[46] Grinnell F. (1984). Fibronectin and Wound Healing. J. Cell. Biochem..

[47] Sgarioto M., Vigneron P., Patterson J., Malherbe F., Nagel M.D., Egles C. (2012). Collagen Type I Together with Fibronectin Provide a Better Support for Endothelialization. Comptes Rendus-Biol..

[48] Ardila D., Liou J.-J., Maestas D., Slepian M., Badowski M., Wagner W., Harris D., Vande Geest J. (2019). Surface Modification of Electrospun Scaffolds for Endothelialization of Tissue-Engineered Vascular Grafts Using Human Cord Blood-Derived Endothelial Cells. J. Clin. Med..

[49] Tersteeg C., Roest M., Mak-Nienhuis E.M., Ligtenberg E., Hoefer I.E., de Groot P.G., Pasterkamp G. (2012). A Fibronectin-Fibrinogen-Tropoelastin Coating Reduces Smooth Muscle Cell Growth but Improves Endothelial Cell Function. J. Cell. Mol. Med..

[50] Ota T., Sawa Y., Iwai S., Kitajima T., Ueda Y., Coppin C., Matsuda H., Okita Y. (2005). Fibronectin-Hepatocyte Growth Factor Enhances Reendothelialization in Tissue-Engineered Heart Valve. Ann. Thorac. Surg..

[51] Wang X., Liu T., Chen Y., Zhang K., Maitz M.F., Pan C., Chen J., Huang N. (2014). Extracellular Matrix Inspired Surface Functionalization with Heparin, Fibronectin and VEGF Provides an Anticoagulant and Endothelialization Supporting Microenvironment. Appl. Surf. Sci..

[52] Hoenig M.R., Campbell G.R., Campbell J.H. (2006). Vascular Grafts and the Endothelium. Endothel. J. Endothel. Cell Res..

[53] Matsuzaki Y., John K., Shoji T., Shinoka T. (2019). The Evolution of Tissue Engineered Vascular Graft Technologies: From Preclinical Trials to Advancing Patient Care. Appl. Sci..

[54] Popov G., Vavilov V., Yukina G., Popryadukhin P., Dobrovolskaya I., Ivan’kova E., Yudin V. (2019). Long-Term Functioning Aneurysmal Free Tissue-Engineered Vascular Graft Based on Composite Bi-Layered (PLLA/FPL) Scaffold. Eur. J. Vasc. Endovasc. Surg..

[55] Kutuzova L., Athanasopulu K., Schneider M., Kandelbauer A., Kemkemer R., Lorenz G. (2018). In Vitro Bio-Stability Screening of Novel Implantable Polyurethane Elastomers: Morphological Design and Mechanical Aspects. Curr. Dir. Biomed. Eng..

[56] Broadwater S.J., Roth S.L., Price K.E., Kobašlija M., McQuade D.T. (2005). One-Pot Multi-Step Synthesis: A Challenge Spawning Innovation. Org. Biomol. Chem..

[57] Athanasopulu K., Kutuzova L., Thiel J., Lorenz G., Kemkemer R. (2019). Enhancing the Biocompatibility of Siliconepolycarbonate Urethane Based Implant Materials. Curr. Dir. Biomed. Eng..

[58] Schindelin J., Arganda-Carreras I., Frise E., Kaynig V., Longair M., Pietzsch T., Preibisch S., Rueden C., Saalfeld S., Schmid B. (2012). Fiji: An Open-Source Platform for Biological-Image Analysis. Nat. Methods.

[59] Laterreur V., Ruel J., Auger F.A., Vallières K., Tremblay C., Lacroix D., Tondreau M., Bourget J.M., Germain L. (2014). Comparison of the Direct Burst Pressure and the Ring Tensile Test Methods for Mechanical Characterization of Tissue-Engineered Vascular Substitutes. J. Mech. Behav. Biomed. Mater..

[60] Hinderer S., Seifert J., Votteler M., Shen N., Rheinlaender J., Schäffer T.E., Schenke-Layland K. (2014). Engineering of a Bio-Functionalized Hybrid off-the-Shelf Heart Valve. Biomaterials.

[61] Becker M., Schneider M., Stamm C., Seifert M. (2019). A Polymorphonuclear Leukocyte Assay to Assess Implant Immunocompatibility. Tissue Eng. Part C Methods.

[62] Schneider M., Stamm C., Brockbank K.G.M., Stock U.A., Seifert M. (2017). The Choice of Cryopreservation Method Affects Immune Compatibility of Human Cardiovascular Matrices. Sci. Rep..

[63] Pusch J., Votteler M., Göhler S., Engl J., Hampel M., Walles H., Schenke-Layland K. (2011). The Physiological Performance of a Three-Dimensional Model That Mimics the Microenvironment of the Small Intestine. Biomaterials.

[64] Piccirillo G., Carvajal Berrio D.A., Laurita A., Pepe A., Bochicchio B., Schenke-Layland K., Hinderer S. (2019). Controlled and Tuneable Drug Release from Electrospun Fibers and a Non-Invasive Approach for Cytotoxicity Testing. Sci. Rep..

[65] Al-Sabti H.A., Al Kindi A., Al-Rasadi K., Banerjee Y., Al-Hashmi K., Al-Hinai A. (2013). Saphenous Vein Graft vs. Radial Artery Graft Searching for the Best Second Coronary Artery Bypass Graft. J. Saudi Heart Assoc..

[66] Stekelenburg M., Rutten M.C.M., Snoeckx L.H.E.H., Baaijens F.P.T. (2009). Dynamic Straining Combined with Fibrin Gel Cell Seeding Improves Strength of Tissue-Engineered Small-Diameter Vascular Grafts. Tissue Eng. Part A.

[67] Soletti L., Hong Y., Guan J., Stankus J.J., El-Kurdi M.S., Wagner W.R., Vorp D.A. (2010). A Bilayered Elastomeric Scaffold for Tissue Engineering of Small Diameter Vascular Grafts. Acta Biomater..

[68] Pukacki F., Jankowski T., Gabriel M., Oszkinis G., Krasinski Z., Zapalski S. (2000). The Mechanical Properties of Fresh and Cryopreserved Arterial Homografts. Eur. J. Vasc. Endovasc. Surg..

[69] Porter T.R., Taylor D.O., Fields J., Cycan A., Akosah K., Mohanty P.K., Pandian N.G. (1993). Direct in Vivo Evaluation of Pulmonary Arterial Pathology in Chronic Congestive Heart Failure with Catheter-Based Intravascular Ultrasound Imaging. Am. J. Cardiol..

[70] Abbott W.M. (1997). Prosthetic Above-Knee Femoral-Popliteal Bypass: Indications and Choice of Graft. Semin. Vasc. Surg..

[71] Konig G., McAllister T.N., Dusserre N., Garrido S.A., Iyican C., Marini A., Fiorillo A., Avila H., Wystrychowski W., Zagalski K. (2009). Mechanical Properties of Completely Autologous Human Tissue Engineered Blood Vessels Compared to Human Saphenous Vein and Mammary Artery. Biomaterials.

[72] Lamm P., Juchem G., Milz S., Schuffenhauer M., Reichart B. (2001). Autologous Endothelialized Vein Allograft: A Solution in the Search for Small-Caliber Grafts in Coronary Artery Bypass Graft Operations. Circulation.

[73] L’Heureux N., Pâquet S., Labbé R., Germain L., Auger F.A. (1998). A Completely Biological Tissue-Engineered Human Blood Vessel. FASEB J..

[74] Caruso A., Licenziati S., Corulli M., Canaris A.D., De Francesco M.A., Fiorentini S., Peroni L., Fallacara F., Dima F., Balsari A. (1997). Flow Cytometric Analysis of Activation Markers on Stimulated T Cells and Their Correlation with Cell Proliferation. Cytometry.

[75] Lerouge S. (2012). Introduction to Sterilization: Definitions and Challenges. Sterilisation of Biomaterials and Medical Devices.

[76] Ameer J.M., Anil Kumar P.R., Kasoju N. (2019). Strategies to Tune Electrospun Scaffold Porosity for Effective Cell Response in Tissue Engineering. J. Funct. Biomater..

[77] Bružauskaitė I., Bironaitė D., Bagdonas E., Bernotienė E. (2016). Scaffolds and Cells for Tissue Regeneration: Different Scaffold Pore Sizes—Different Cell Effects. Cytotechnology.

[78] Murphy C.M., O’Brien F.J. (2010). Understanding the Effect of Mean Pore Size on Cell Activity in Collagen-Glycosaminoglycan Scaffolds. Cell Adhes. Migr..

[79] Arends F., Lieleg O. (2016). Biophysical Properties of the Basal Lamina: A Highly Selective Extracellular Matrix. Composition and Function of the Extracellular Matrix in the Human Body.

[80] Liliensiek S.J., Nealey P., Murphy C.J. (2009). Characterization of Endothelial Basement Membrane Nanotopography in Rhesus Macaque as a Guide for Vessel Tissue Engineering. Tissue Eng. Part A.

[81] Sage H. (1982). Collagens of Basement Membranes. J. Investig. Dermatol..

[82] Abrams G.A., Murphy C.J., Wang Z.Y., Nealey P.F., Bjorling D.E. (2003). Ultrastructural Basement Membrane Topography of the Bladder Epithelium. Urol. Res..

[83] Inoguchi H., Kwon I.K., Inoue E., Takamizawa K., Maehara Y., Matsuda T. (2006). Mechanical Responses of a Compliant Electrospun Poly(L-Lactide-Co-ε- Caprolactone) Small-Diameter Vascular Graft. Biomaterials.

[84] Nottelet B., Pektok E., Mandracchia D., Tille J.-C., Walpoth B., Gurny R., Möller M. (2009). Factorial Design Optimization and *in Vivo* Feasibility of Poly(ε-Caprolactone)-Micro- and Nanofiber-Based Small Diameter Vascular Grafts. J. Biomed. Mater. Res. Part A.

[85] Vaz C.M., van Tuijl S., Bouten C.V.C., Baaijens F.P.T. (2005). Design of Scaffolds for Blood Vessel Tissue Engineering Using a Multi-Layering Electrospinning Technique. Acta Biomater..

[86] Hironaka K., Makino H., Yamasaki Y., Ota Z. (1993). Renal Basement Membranes by Ultrahigh Resolution Scanning Electron Microscopy. Kidney Int..

[87] Takeuchi T., Gonda T. (2004). Distribution of the Pores of Epithelial Basement Membrane in the Rat Small Intestine. J. Vet. Med. Sci..

[88] Yurchenco P.D., Ruben G.C. (1987). Basement Membrane Structure in Situ: Evidence for Lateral Associations in the Type IV Collagen Network. J. Cell Biol..

[89] Howat W.J., Holmes J.A., Holgate S.T., Lackie P.M. (2001). Basement Membrane Pores in Human Bronchial Epithelium: A Conduit for Infiltrating Cells?. Am. J. Pathol..

[90] Wierzbicka-Patynowski I., Schwarzbauer J.E. (2003). Cell-Surface Transglutaminase Promotes Fibronectin Assembly via Interaction with the Gelatin-Binding Domain of Fibronectin: A Role in TGFbeta-Dependent Matrix Deposition. J. Cell Sci..

[91] Sevilla C.A., Dalecki D., Hocking D.C. (2013). Regional Fibronectin and Collagen Fibril Co-Assembly Directs Cell Proliferation and Microtissue Morphology. PLoS ONE.

[92] Rico P., Mnatsakanyan H., Dalby M.J., Salmerón-Sánchez M. (2016). Material-Driven Fibronectin Assembly Promotes Maintenance of Mesenchymal Stem Cell Phenotypes. Adv. Funct. Mater..

[93] Llopis-Hernández V., Cantini M., González-García C., Cheng Z.A., Yang J., Tsimbouri P.M., García A.J., Dalby M.J., Salmerón-Sánchez M. (2016). Material-Driven Fibronectin Assembly for High-Efficiency Presentation of Growth Factors. Sci. Adv..

[94] Schmidt G., Hausser H., Kresse H. (1991). Interaction of the Small Proteoglycan Decorin with Fibronectin. Involvement of the Sequence NKISK of the Core Protein. Biochem. J..

[95] Winnemöller M., Schmidt G., Kresse H. (1991). Influence of Decorin on Fibroblast Adhesion to Fibronectin. Eur. J. Cell Biol..

[96] Dee K.C., Puleo D.A., Bizios R. (2002). Protein-Surface Interactions. An Introduction to Tissue-Biomaterial Interaction.

[97] Thyparambil A.A., Wei Y., Latour R.A. (2015). Experimental Characterization of Adsorbed Protein Orientation, Conformation, and Bioactivity. Biointerphases.

[98] Latour R.A., Puleo D.A., Bizios R. (2009). Molecular Simulation of Protein-Surface Interactions. Biological Interactions on Material Surfaces.

[99] Lebaron R.G., Athanasiou K.A. (2000). Extracellular Matrix Cell Adhesion Peptides: Functional Applications in Orthopedic Materials. Tissue Eng..

[100] Hinderer S., Schesny M., Bayrak A., Ibold B., Hampel M., Walles T., Stock U.A., Seifert M., Schenke-Layland K. (2012). Engineering of Fibrillar Decorin Matrices for a Tissue-Engineered Trachea. Biomaterials.

[101] Ishizaki T., Saito N., Takai O. (2010). Correlation of Cell Adhesive Behaviors on Superhydrophobic, Superhydrophilic, and Micropatterned Superhydrophobic/Superhydrophilic Surfaces to Their Surface Chemistry. Langmuir.

[102] Schmidt G., Robenek H., Harrach B., Glössl J., Nolte V., Hörmann H., Richter H., Kresse H. (1987). Interaction of Small Dermatan Sulfate Proteoglycan from Fibroblasts with Fibronectin. J. Cell Biol..

[103] Zhao J., Mitrofan C.G., Appleby S.L., Morrell N.W., Lever A.M.L. (2016). Disrupted Endothelial Cell Layer and Exposed Extracellular Matrix Proteins Promote Capture of Late Outgrowth Endothelial Progenitor Cells. Stem Cells Int..

[104] Brown B.N., Badylak S.F. (2013). Expanded Applications, Shifting Paradigms and an Improved Understanding of Host-Biomaterial Interactions. Acta Biomater..

[105] Wang Z., Cui Y., Wang J., Yang X., Wu Y., Wang K., Gao X., Li D., Li Y., Zheng X.L. (2014). The Effect of Thick Fibers and Large Pores of Electrospun Poly(ε-Caprolactone) Vascular Grafts on Macrophage Polarization and Arterial Regeneration. Biomaterials.

[106] Dejana E., Orsenigo F., Molendini C., Baluk P., McDonald D.M. (2009). Organization and Signaling of Endothelial Cell-to-Cell Junctions in Various Regions of the Blood and Lymphatic Vascular Trees. Cell Tissue Res..

[107] Lertkiatmongkol P., Liao D., Mei H., Hu Y., Newman P.J. (2016). Endothelial Functions of Platelet/Endothelial Cell Adhesion Molecule-1 (CD31). Curr. Opin. Hematol..

[108] Yamaguchi T.P., Dumont D.J., Conlon R.A., Breitman M.L., Rossant J. (1993). Flk-1, an Fit-Related Receptor Tyrosine Kinase Is an Early Marker for Endothelial Cell Precursors. Development.

[109] Smadja D.M., Bièche I., Helley D., Laurendeau I., Simonin G., Muller L., Aiach M., Gaussem P. (2007). Increased VEGFR2 Expression during Human Late Endothelial Progenitor Cells Expansion Enhances in Vitro Angiogenesis with Up-Regulation of Integrin A6. J. Cell. Mol. Med..

[110] Kusuma S., Zhao S., Gerecht S. (2012). The Extracellular Matrix Is a Novel Attribute of Endothelial Progenitors and of Hypoxic Mature Endothelial Cells. FASEB J..

[111] Ichii M., Frank M.B., Iozzo R.V., Kincade P.W. (2012). The Canonical Wnt Pathway Shapes Niches Supportive of Hematopoietic Stem/Progenitor Cells. Blood.

[112] Wijelath E.S., Rahman S., Murray J., Patel Y., Savidge G., Sobel M. (2004). Fibronectin Promotes VEGF-Induced CD34+ Cell Differentiation into Endothelial Cells. J. Vasc. Surg..

[113] Shen B.Q., Lee D.Y., Gerber H.P., Keyt B.A., Ferrara N., Zioncheck T.F. (1998). Homologous Up-Regulation of KDR/Flk-1 Receptor Expression by Vascular Endothelial Growth Factor in Vitro. J. Biol. Chem..

[114] Neill T., Schaefer L., Iozzo R.V. (2012). Decorin: A Guardian from the Matrix. Am. J. Pathol..

[115] Mazor R., Alsaigh T., Shaked H., Altshuler A.E., Pocock E.S., Kistler E.B., Karin M., Schmid-Schönbein G.W. (2013). Matrix Metalloproteinase-1-Mediated up-Regulation of Vascular Endothelial Growth Factor-2 in Endothelial Cells. J. Biol. Chem..

[116] Huttenlocher A., Werb Z., Tremble P., Huhtala P., Rosenberg L., Damsky C.H. (1996). Decorin Regulates Collagenase Gene Expression in Fibroblasts Adhering to Vitronectin. Matrix Biol..

[117] Schönherr E., Schaefer L., O’Connell B.C., Kresse H. (2001). Matrix Metalloproteinase Expression by Endothelial Cells in Collagen Lattices Changes during Co-Culture with Fibroblasts and upon Induction of Decorin Expression. J. Cell. Physiol..

[118] Murakami M., Nguyen L.T., Hatanaka K., Schachterle W., Chen P.Y., Zhuang Z.W., Black B.L., Simons M. (2011). FGF-Dependent Regulation of VEGF Receptor 2 Expression in Mice. J. Clin. Investig..

[119] Penc S.F., Pomahac B., Winkler T., Dorschner R.A., Eriksson E., Herndon M., Gallo R.L. (1998). Dermatan Sulfate Released after Injury Is a Potent Promoter of Fibroblast Growth Factor-2 Function. J. Biol. Chem..

[120] Chen T.T., Filvaroff E., Peng J., Marsters S., Jubb A., Koeppen H., Merchant M., Ashkenazi A. (2015). MET Suppresses Epithelial VEGFR2 via Intracrine VEGF-Induced Endoplasmic Reticulum-Associated Degradation. EBioMedicine.

[121] Chen S., Chakrabarti R., Keats E.C., Chen M., Chakrabarti S., Khan Z.A. (2012). Regulation of Vascular Endothelial Growth Factor Expression by Extra Domain B Segment of Fibronectin in Endothelial Cells. Investig. Ophthalmol. Vis. Sci..

[122] Wijelath E.S., Rahman S., Namekata M., Murray J., Nishimura T., Mostafavi-Pour Z., Patel Y., Suda Y., Humphries M.J., Sobel M. (2006). Heparin-II Domain of Fibronectin Is a Vascular Endothelial Growth Factor-Binding Domain. Circ. Res..

[123] Kou R., SenBanerjee S., Jain M.K., Michel T. (2005). Differential Regulation of Vascular Endothelial Growth Factor Receptors (VEGFR) Revealed by RNA Interference: Interactions of VEGFR-1 and VEGFR-2 in Endothelial Cell Signaling. Biochemistry.

[124] Scott R.A., Paderi J.E., Sturek M., Panitch A. (2013). Decorin Mimic Inhibits Vascular Smooth Muscle Proliferation and Migration. PLoS ONE.

[125] Harding A., Cortez-Toledo E., Magner N.L., Beegle J.R., Coleal-Bergum D.P., Hao D., Wang A., Nolta J.A., Zhou P. (2017). Highly Efficient Differentiation of Endothelial Cells from Pluripotent Stem Cells Requires the MAPK and the PI3K Pathways. Stem Cells.

[126] Bryan B.A., Walshe T.E., Mitchell D.C., Havumaki J.S., Saint-Geniez M., Maharaj A.S., Maldonado A.E., D’Amore P.A. (2008). Coordinated Vascular Endothelial Growth Factor Expression and Signaling during Skeletal Myogenic Differentiation. Mol. Biol. Cell.

[127] Zeng Y., Zhang X.F., Fu B.M., Tarbell J.M. (2018). The Role of Endothelial Surface Glycocalyx in Mechanosensing and Transduction. Advances in Experimental Medicine and Biology.

[128] Ballermann B.J., Dardik A., Eng E., Liu A. (1998). Shear Stress and the Endothelium. Kidney Int..

[129] Sato M., Kataoka N., Ohshima N. (1996). Response of Vascular Endothelial Cells to Flow Shear Stress: Phenomenological Aspect. Biomechanics.

[130] Zhou J., Li Y.S., Chien S. (2014). Shear Stress-Initiated Signaling and Its Regulation of Endothelial Function. Arterioscler. Thromb. Vasc. Biol..

[131] Sánchez-Duffhues G., García de Vinuesa A., ten Dijke P. (2018). Endothelial-to-Mesenchymal Transition in Cardiovascular Diseases: Developmental Signaling Pathways Gone Awry. Dev. Dyn..

[132] Moonen J.R.A.J., Lee E.S., Schmidt M., Maleszewska M., Koerts J.A., Brouwer L.A., Van Kooten T.G., Van Luyn M.J.A., Zeebregts C.J., Krenning G. (2015). Endothelial-to-Mesenchymal Transition Contributes to Fibro-Proliferative Vascular Disease and Is Modulated by Fluid Shear Stress. Cardiovasc. Res..

[133] Mahmoud M.M., Serbanovic-Canic J., Feng S., Souilhol C., Xing R., Hsiao S., Mammoto A., Chen J., Ariaans M., Francis S.E. (2017). Shear Stress Induces Endothelial-To-Mesenchymal Transition via the Transcription Factor Snail. Sci. Rep..

[134] Melchiorri A.J., Bracaglia L.G., Kimerer L.K., Hibino N., Fisher J.P. (2016). In Vitro Endothelialization of Biodegradable Vascular Grafts Via Endothelial Progenitor Cell Seeding and Maturation in a Tubular Perfusion System Bioreactor. Tissue Eng.-Part C Methods.

[135] Dejana E., Hirschi K.K., Simons M. (2017). The Molecular Basis of Endothelial Cell Plasticity. Nat. Commun..

[136] Lacorre D.A., Baekkevold E.S., Garrido I., Brandtzaeg P., Haraldsen G., Amalric F., Girard J.P. (2004). Plasticity of Endothelial Cells: Rapid Dedifferentiation of Freshly Isolated High Endothelial Venule Endothelial Cells Outside the Lymphoid Tissue Microenvironment. Blood.

[137] Nguyen M.T.X., Okina E., Chai X., Tan K.H., Hovatta O., Ghosh S., Tryggvason K. (2016). Differentiation of Human Embryonic Stem Cells to Endothelial Progenitor Cells on Laminins in Defined and Xeno-Free Systems. Stem Cell Rep..

