# Supplementary material for: Fibronectin Adsorption on Electrospun Synthetic Vascular Grafts Attracts Endothelial Progenitor Cells and Promotes Endothelialization in Dynamic In Vitro Culture
Source: Cells. 2020 Mar 23;9(3):778. doi: 10.3390/cells9030778 (PMC7140838; doi:10.3390/cells9030778)
Supplement: Supplementary file 1 [file cells-09-00778-s001.pdf]

Supplementary Materials

1. Mechanical characterization of the electrospun TPCU scaffold

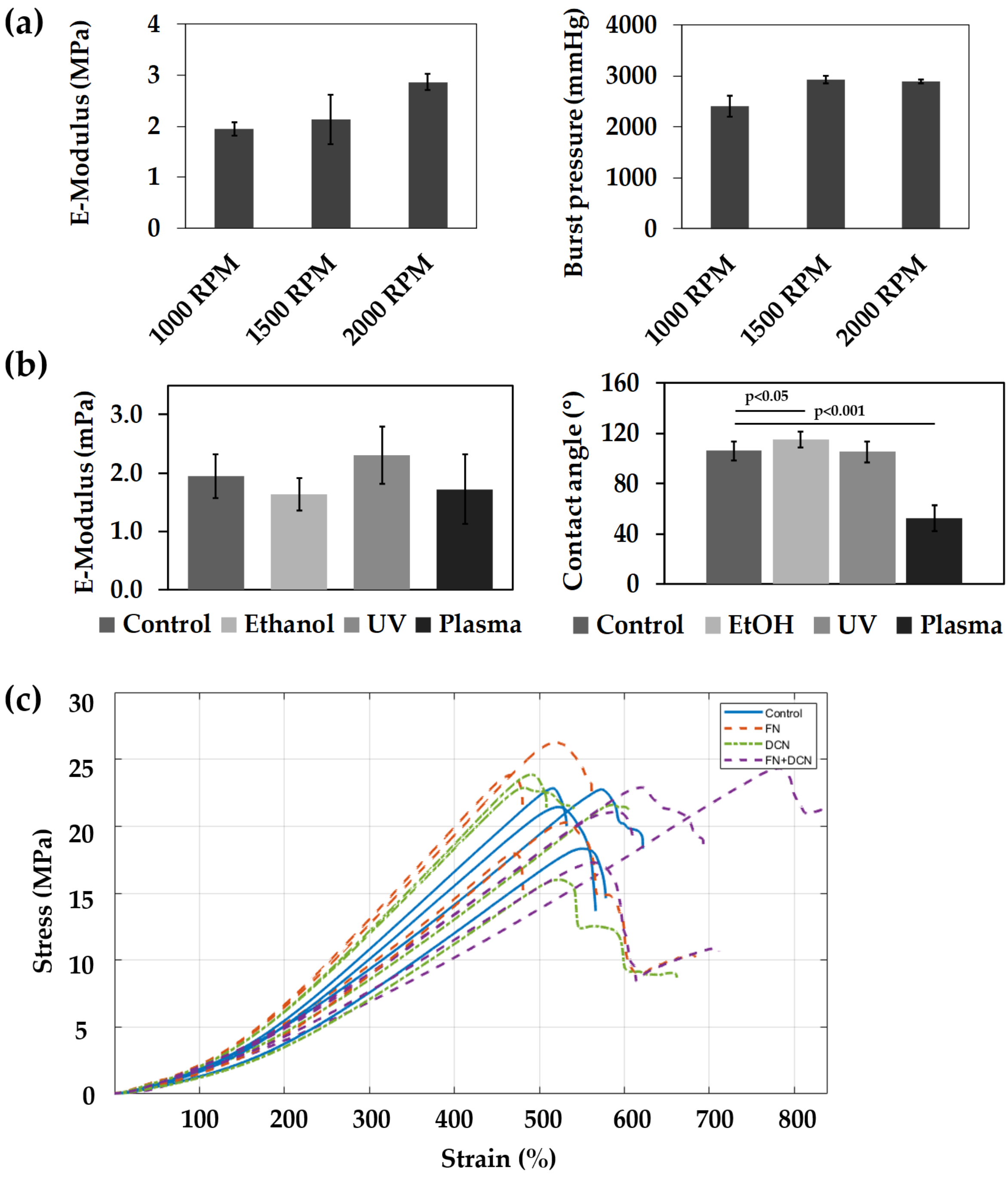

Figure S1. Mechanical characterization of the electrospun TPCU scaffold and biocompatibility of the materials. (**a**) E-modulus and burst pressure of tubular constructs spun with different mandrel rotating speeds. (**b**) Wettability and E-modulus of 70 % ethanol, UV and oxygen plasma treated electrospun TPCU scaffolds. Two-tailed *t*-test, n=3 (**c**) Stress-strain curves of control, FN, DCN and FN+DCN coated scaffolds.

2. Cytotoxicity of the materials used in the study

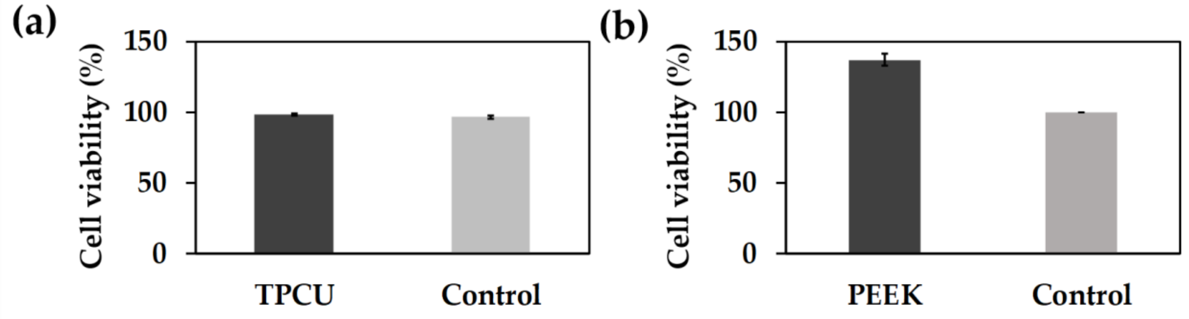

Figure S2. Cytotoxicity tests of the materials. Both (**a**) TPCU and (**b**) PEEK (used in the bioreactor) showed no cytotoxic effects.

3. Microbiological studies of the electrospun scaffolds

Method

In order to investigate the disinfection method, microbiological studies of the electrospun scaffolds were performed. For this approach, nine round TPCU scaffolds with a diameter of 3 cm were disinfected with 70% ethanol for 20 min. After washing three times with PBS under sterile conditions, each scaffold was transferred into 50 ml LB-medium (1% tryptone, 0.5% yeast extract, 1% sodium chloride in H_2O) and incubated for 48 hours at 37 °C. The medium without the scaffold served as a control. Subsequently, 0.1 mL of the LB-medium were plated on agar plates (4% LB-agar, Carl Roth, Karlsruhe, Germany) and incubated over night at 37 °C and 90% humidity. Finally, the germ load on the agar plates was examined macroscopically.

Results and Discussion

Two of the nine scaffolds showed a bacterial contamination after ethanol treatment (**Figure S3**). This result shows that our disinfection method does not guarantee 100% sterility. However, we are aware that ethanol treatment as a disinfection method does not necessarily inactivate all forms of microorganisms [1]. In order to take this into account in our study, penicillin-streptomycin was added to all cell culture experiments to suppress possible contamination. In a next step, sterilization methods should be investigated, such as gamma irradiation or ethylene oxide treatment.

(a)

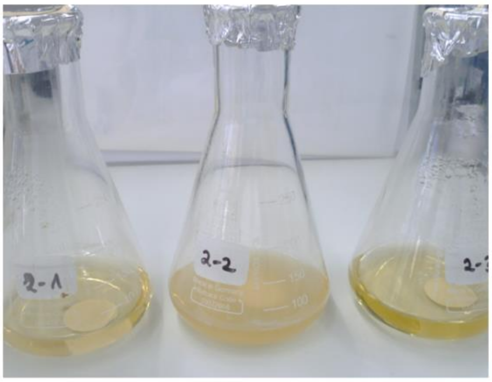

(b)

sample	CFU*/mL	germ load
negative control	0	negative
1	0	negative
2	0	negative
3	0	negative
4	0	negative
5	>300	positive
6	0	negative
7	0	negative
8	>300	positive
9	0	negative

*CFU = Colony Forming Unit

(c)

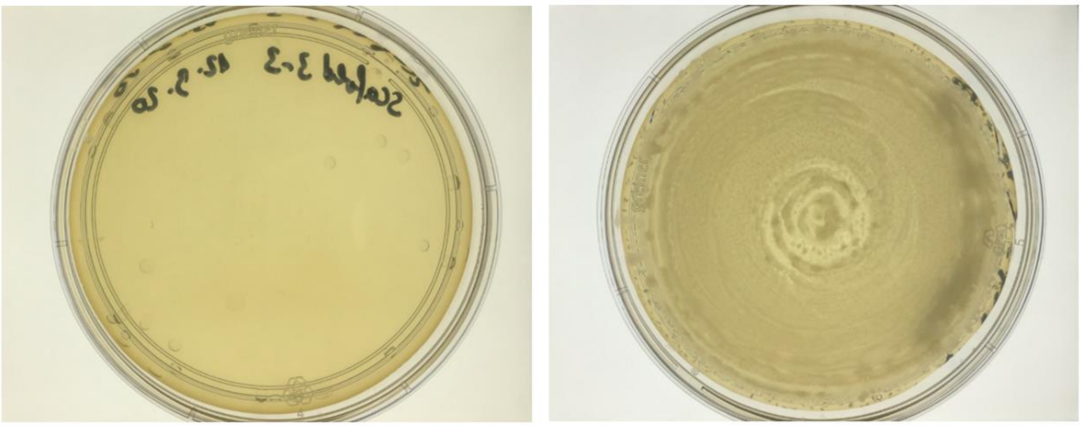

Figure S3. Microbiological studies of the ethanol disinfected electrospun TPCU scaffolds (**a**) Scaffolds were incubated in LB-medium for 48 hours at 37 °C. (**b**) In terms of germ load, two of nine scaffolds were positive. CFU stands for colony forming unit. (**c**) Macroscopic image of a germ-free (left) and a germ-contaminated (right) agar plate.

4. In silico CFD simulations

Methods

The CAD models were designed in Solidworks and consequently prepared for numerical fluid simulations. The three-dimensional surfaces of the bioreactor culture chamber model, which are in contact with the intraluminal medium, were imported into ANSYS Mesher, whilst omitting other irrelevant parts of the culturing chamber. The result practically resembled a 'pipe model' of the intraluminal circulation, with scaffold diameters ranging between 3.0 and 6.0 mm (**Error! Reference source not found.**). To assure a quicker convergence and stable results, the thee-dimensional pipe model was mostly meshed with a hexahedronal (six-sided) mesh structure. The resulting cell count in the meshed models was around 500.000 cells. The meshes were imported in Fluent 19, executed in the three-dimensional double precision mode and appointed all eight logical CPU cores on the computer. The simulation was carried out with a rigid model of the scaffold wall. A paraboloid velocity distribution was imposed on the inlet, representing an already fully developed flow profile at the entrance of the culture chamber. Furthermore, a no-slip velocity boundary condition was imposed on the walls. The numerical simulations in ANSYS Fluent were performed using the built-in pressure-based

solver and the second-order upwind momentum discretization scheme. Cell culture medium was approximated to have the same rheological properties as water at 37 °C, with a dynamic viscosity of 0.691 mPa s and a density of 993 kg m^{-3}. CFD simulations were carried out for a range of flow rates between 0.2 and 50.0 mL/min.

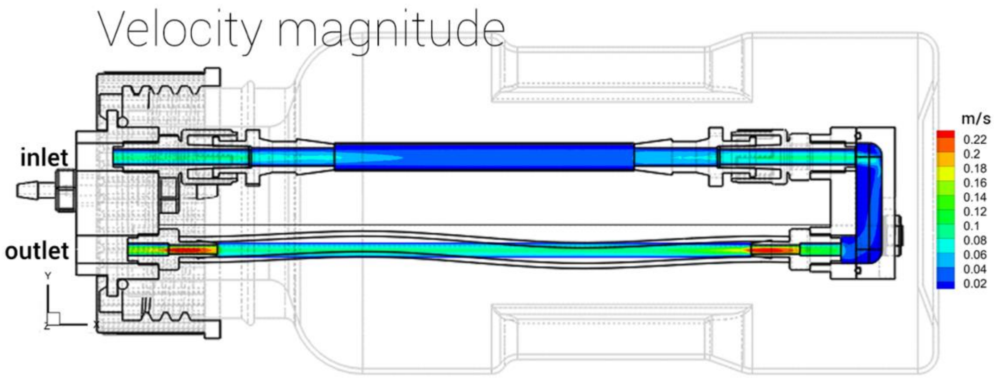

Figure S4. The part of the culture chamber that was considered for CFD simulations. Different flow rates and scaffold diameters were considered for which the geometry was adapted accordingly.

Results

The flow regime was analyzed and was found to be laminar within the operational range. The wall shear stress along the inner scaffold wall for different flow rates are plotted in **Error! Reference source not found.**. The observed Poiseuille values in the CFD simulations corresponded well to the analytical solution for the wall shear stress of a laminar flow in straight circular pipe according to the Hagen-Poiseuille equation for all flow rates between 0.2 and 20 ml/min.

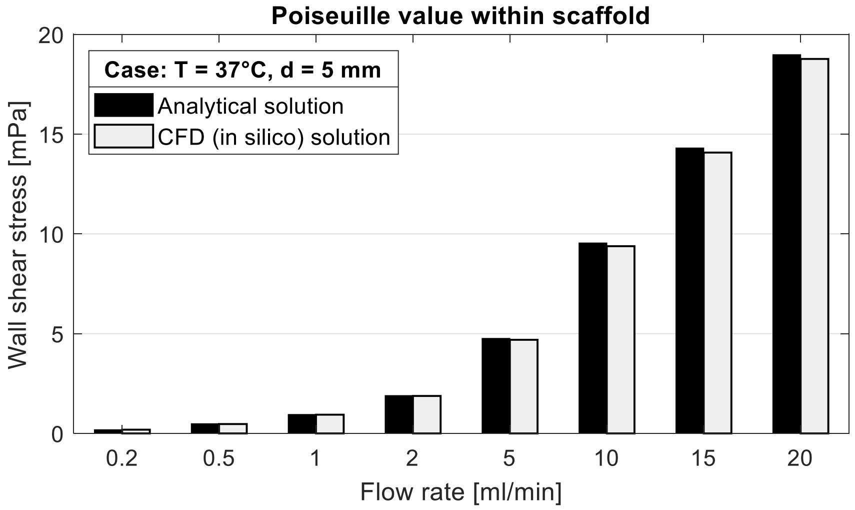

Figure S5. The Poiseuille values (developed wall shear stress value) within the scaffold for different flow rates. This plot compares the observed Poiseuille values to those of the analytical solution.

1. Lerouge, S. Introduction to Sterilization: Definitions and Challenges. In *Sterilisation of Biomaterials and Medical Devices*; Elsevier, **2012**; pp. 1–19.
